# Synthesis and Characterization of Functionalized Polylactides Containing Acetal Units

**DOI:** 10.1021/acs.macromol.3c01343

**Published:** 2023-08-16

**Authors:** Karolina Cichoń, Irena I. Bak-Sypien, Malgorzata Basko, Bartłomiej Kost

**Affiliations:** Centre of Molecular and Macromolecular Studies Polish Academy of Sciences Sienkiewicza 112, 90-363 Lodz, Poland

## Abstract

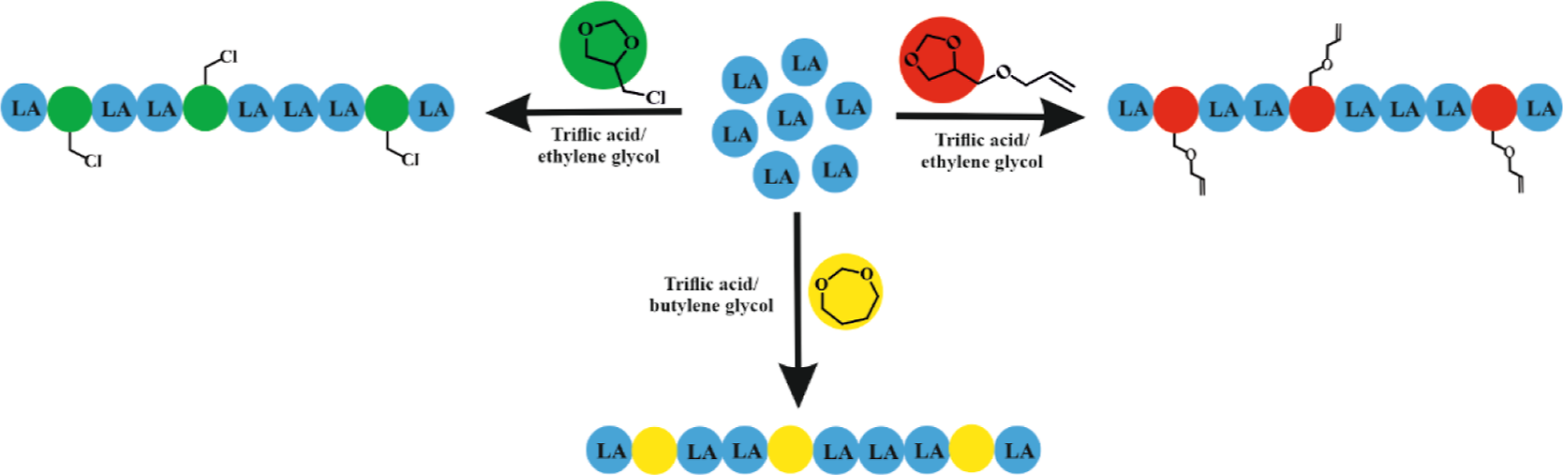

New functionalized lactide copolymers containing acetal units were prepared for the first time in a controlled manner that enabled the regulation of the number of reactive groups introduced into the polyester chain. The presence of functional groups in the copolymer backbone provided chemical modification sites, and the nature of the acetal unit affected the material degradability. First, paraformaldehyde was reacted with selected diols containing reactive pendant groups (3-allyloxypropane-1,2-diol and 3-chloropropane-1,2-diol), which was catalyzed by *p*-toluenesulfonic acid, to synthesize new cyclic acetals with different functionalities (allyl- or chloro-). In addition, using butane-1,4-diol, a nonfunctionalized seven-membered cyclic acetal (dioxepane) was obtained for comparative studies. In the next step, the prepared cyclic acetals were used for cationic copolymerization with lactide in the presence of glycol as an initiator and triflic acid as a catalyst. Different temperatures (−15, 2, and 30 °C) and copolymerization times (24, 48, 72, and 192 h) were investigated to produce copolyesters with variable contents of acetal units in the range of 5–27%. The copolymers’ structure and molar masses were carefully investigated using ^1^H, ^13^C NMR, 2D NMR, and size-exclusion chromatography. Moreover, the ability of functionalized copolymers to perform post modifications was also proven by the reaction with sodium azide and propanethiol. Finally, we speculate that structurally diverse groups can be attached to the copolyester chain, fine-tuning the on-demand properties, which could rapidly expand the library of polylactide-based materials.

## Introduction

Research on the synthesis of biodegradable polymers used in various fields, such as medicine, health care, packaging, or environmental protection, is increasingly important in polymer science.^1^ Currently, several different types of biodegradable polymers have been developed as potential “green materials” due to their specific properties, which can be adapted to the intended applications.^2,3^ Among them, polyesters, such as polylactide, polycaprolactone, polyglycolide, or their copolymers, are the most promising candidates to replace nondegradable petroleum-based polymers. Due to its biocompatibility, biodegradability, and the possibility of obtaining raw materials for synthesis from renewable sources, polylactide (PLA) may be the most promising eco-friendly polymer with great potential for further development.^4^ However, PLA also has numerous disadvantages that limit its application. For instance, to achieve sufficient degradation time, the molecular weight of PLA should be lower than 40 000 g/mol; otherwise, the polyester can degrade even for 60 years.^5^ On the other hand, in the case of packaging applications, its relatively high crystallinity and brittleness may cause the material properties to deteriorate. The difficulty of modifying PLA is among the most important drawbacks as the linear aliphatic structure of the repeating unit does not contain any reactive groups in the polymer chain. The lack of functional side groups in polylactide strongly limits the possibility of subsequent post modification through further reactions with purposely selected compounds. Therefore, much scientific research has focused on finding new methods and approaches for the functionalization of polylactide.^6^ A widely used way to modify the chemical properties of polylactide is the copolymerization of lactide with different monomers that may introduce degradable or nondegradable units into the polyester chain.

One of the first copolymers, which presented attractive mechanical and processing properties suitable for medical applications, was obtained in the copolymerization of lactide with glycolide.^7,8^ Other cyclic monomers such as caprolactone,^9^ valerolactone,^10^ trimethylene carbonate,^11^ or ethylene oxide^12^ were also copolymerized with lactide to obtain a better balance of properties of the polymeric material. These comonomers enable the crystallinity, hydrophobicity, and mechanical properties of the material to be changed; however, they do not enable the functionalization of the polyester chain.^13^ To obtain polyesters containing reactive groups in the side chains, functionalized comonomers were used in the copolymerization, which allowed polylactide with various groups, such as hydroxyl,^14^ vinyl, allyl,^15^ propargyl,^16^ and chloromethylene^17^ groups, to be synthesized. However, very often, introducing these pendant reactive groups into the polyester chain involves inserting repeating units (e.g., allyl and propargyl glycidyl ethers) that do not significantly improve the degradation rate of the final copolymer. Moreover, in some cases, only a few units of functional comonomer can be incorporated into the polymer chain (for example, in the copolymerization of lactide with substituted ethers, functional copolymers containing several functionalized units per chain were obtained).^16,18^ When copolymerizing lactide with substituted lactides, multistage synthesis, which often proceeds with a low efficiency, must be considered.^17^ Moreover, to incorporate the functionalities that can initiate polymerization (amine or hydroxyl groups), the lactide must first be blocked by a protecting group (e.g., benzyl group) in the monomer to prevent branched or cross-linked structures from forming. Thus, this process requires an additional step after polymerization to unblock the functionalities.^14^ Due to these limitations, further studies on the functionalization of polyesters are justified.

Currently, one of the research directions in polymer chemistry is focused on the synthesis of polyesters containing unstable acetal repeating units in the main chain in order to obtain hydrolytically and thermally sensitive polymers. Various methods can achieve this effect. For example, cyclic hemiacetal esters, which are more hydrolytically and thermally labile than their acetal and ester counterparts, were used in cationic ring-opening polymerization enabling efficient synthesis of polymers with linkages that facilitated degradation.^19–21^

Our research group is performing extensive research to develop polyesters with modified properties by ring-opening copolymerization of lactide with cyclic acetals.^22,23^ We determined that copolymerization with 1,3-dioxolane allowed for the crystallinity and degradation rate of the resulting polylactide to be modified. Labile acetal units in the polylactide copolymers cause material degradation in the presence of the acid for 72 h.^22^ However, despite the accelerated degradation of the copolymer, 1,3-dioxolane does not introduce any additional functional groups that can create the possibility of further postmodification.

The aim of this work was to investigate the copolymerization of lactide with functionalized cyclic acetals to afford copolyesters in which different reactive pendant groups are distributed along the copolymer chain. To achieve this goal, cyclic acetals with allyl- or chloro-functional groups were copolymerized with lactide in the presence of diols (initiators) and strong protonic acid as a catalyst. Basic relationships between the reaction temperature, time, molar mass, and copolymer composition were established. The content of functional groups and acetal units and the structure of the copolymer chain were confirmed by ^1^H, ^13^C NMR, and 2D NMR. Moreover, the ability to functionalize copolymers was also proven by reaction with sodium azide, glycidyl propargyl ether, propargyl alcohol, mercaptoethanol, thioglycolic acid, and propanethiol. We anticipated that through our approach, functional copolyesters containing “clickable” pendant groups can be synthesized with an enhanced degradation ability.

## Experimental Section

### Materials

l,l-Lactide (LA) was purchased from Purac (99% Netherlands), crystallized from 2-propanol and stored under reduced pressure. Paraformaldehyde (99%), *p*-toluenesulfonic acid (pTSA, 98%), 3-allyloxypropane-1,2-diol (98%), 3-chloropropane-1,2-diol (98%), triflic acid (99%), phosphorus pentoxide (P_2_O_5_), propanethiol (99%), mercaptoethanol (95%), thioglycolic acid (98%), propargyl glycidyl ether (98%), propargyl alcohol (97%), copper (I) bromide, pentamethyldiethylenetriamine (95%, PMDTA), and sodium azide were purchased from Merck and used without further purification. Butan-1,4-diol (99%, Acros) was used as received. AIBN was purchased from Sigma-Aldrich and crystallized from ethanol at 40 °C. Methylene chloride from POCH was distilled over P_2_O_5_ and maintained under a vacuum. Toluene (95%), hexane (95%), diethyl ether (98%), and HCl (35%) were purchased from POCH (Gliwice, Poland) and used as received. Anhydrous ethylene glycol (ethane-1,2-diol) (<99.9%, Sigma-Aldrich) was used as received.

### Synthesis of 1,3-Dioxepane (DXP)

50 g of butan-1,4-diol (0.55 mol), 20 g (0.71 mol) of paraformaldehyde, 94 mg (0,55 mmol) of pTSA, and 80 mL of toluene were placed in a round-bottomed flask. Then, the Dean–Stark apparatus and reflux condenser were placed in the flask, and the mixture was refluxed. The synthesis was performed until the appropriate amount of water was released (4 h). Subsequently, the obtained dioxepane was distilled (115–118 °C) under atmospheric pressure with a Vigreux column (40 cm) to well separate toluene (110 °C) from the product. The product with a purity of >99% (GC) was obtained with a yield of 90%. The product was kept over dried molecular sieves (4 Å) under an argon atmosphere.

^1^H NMR, CDCl_3_, 400 MHz, δ (ppm): 4.83 (2H, s, –OCH_2_O−), 3.75 (4H, s, −CH_2_OCH_2_CH_2_OCH_2_−), 1.73 (4H, s, –CH_2_CH_2_OCH_2_CH_2_–).

^13^C NMR, CDCl_3_, 100 MHz, δ (ppm): 94.48 (1C, s, –OCH_2_O−), 67.28 (2C, s, −CH_2_OCH_2_CH_2_OCH_2_−), 29.35 (2C, s, –CH_2_CH_2_OCH_2_CH_2_–).

MS: EI (70 eV), *m*/*z* (%): 102.00 [M]^+^ (11), 71.15 (100), 55.20 (13), 42.25 (93); CI (CH_4_), *m*/*z* calcd, 103.07; found, 103.05 [MH]^+^.

### Synthesis of 4-Chloromethyl-1,3-dioxolane (Cl-DXL)

The synthesis of Cl-DXL was conducted as performed for DXP. Briefly, 50 g of 3-chloropropane-1,2-diol (0.45 mol), 15 g (0.54 mol) of paraformaldehyde, 77 mg (0,45 mmol) of pTSA, and 80 mL of toluene were placed in a round-bottomed flask. Then, the Dean–Stark apparatus and reflux condenser were placed in the flask, and the mixture was refluxed. Then, the toluene was removed from the product by using a rotary evaporator, and the crude Cl-DXL was distilled (146–150 °C) under atmospheric pressure. The product with a purity of >99% (GC) was obtained with a yield of 80%. The product was kept over dried molecular sieves (4 Å) under an argon atmosphere.

^1^H NMR, CDCl_3_, 400 MHz, δ (ppm): 5.07 and 4.90 (2H, s, –OCH_2_O−), 4.31–4.24 (1H, m, –OCH_2_O–CH(CH_2_Cl)CH_2_), 4.02–3.81 (2H, m, –OCH_2_O–CH(CH_2_Cl)CH_2_), 3.62–3.44 (2H, m, –OCH_2_O–CH(CH_2_Cl)CH_2_).

^13^C NMR, CDCl_3_, 100 MHz, δ (ppm): 95.62 (1C, s, –OCH_2_O−), 74.75 (1C, s, –OCH_2_O–CH(CH_2_Cl)CH_2_), 67.90 (1C, s, –OCH_2_O–CH(CH_2_Cl)CH_2_), 44.03 (1C, s, –OCH_2_O–CH(CH_2_Cl)CH_2_).

MS: EI (70 eV), *m*/*z* (%): 121.00 [M-H]^+^ (20), 73.05 (100), 57.00 (10), 45.05 (36); CI (CH_4_), *m*/*z* calcd, 139.05; found, 138.95 [MH]^+^ + CH_4_.

### Synthesis of 4-[(Allyloxy)methyl]-1,3-dioxolane (Allyl-DXL)

50 g of 3-propoxypropane-1,2-diol (0.39 mol), 15 g (0.53 mol) of paraformaldehyde, 68 mg (0,39 mmol) of pTSA, and 80 mL of toluene were placed in a round-bottomed flask. Then, the Dean–Stark apparatus and reflux condenser were placed in the flask. The synthesis was performed until an appropriate amount of water was released. Then, the toluene was removed from the product by using a rotary evaporator, and the crude Allyl-DXL was distilled (45–50 °C) under reduced pressure (1 × 10^–1^ mbar). The product with a purity of >99% (GC) was obtained with a yield of 85%. The product was kept over dried molecular sieves (4 Å) under an argon atmosphere at 4 °C.

^1^H NMR, CDCl_3_, 400 MHz, δ (ppm): 5.97–5.86 (1H, m, CH_2_=CH–CH_2_–O−), 5.35–5.17 (2H, m CH_2_=CH–CH_2_–O−), 5.07 and 4.90 (2H, s, –OCH_2_O−), 4.29–4.20 (1H, quin, –OCH_2_O–CH(CH_2_O−)CH_2_), 4.05 (2H, dd, CH_2_=CH–CH_2_–O−), 3.98 and 3.72 (2H, m, –OCH_2_O–CH(CH_2_O−)CH_2_), 3.58–3.46 (2H, m, CH_2_=CH–CH_2_–O–CH_2_–).

^13^C NMR, CDCl_3_, 100 MHz, δ (ppm): 134.37 (1C, m, CH_2_=CH–CH_2_–O−), 117.14 (1C, m CH_2_=CH–CH_2_–O−), 95.16 (1C, s, –OCH_2_O−), 74.29 (1C, s, –OCH_2_O–CH(CH_2_O−)CH_2_), 72.36 (1C, dd, CH_2_=CH–CH_2_–O−), 70.25 (1C, m, –OCH_2_O–CH(CH_2_O−)CH_2_), 66.91 (1C, m, CH_2_=CH–CH_2_–O–CH_2_–).

MS: EI (70 eV), *m*/*z* (%): 143 [M-H]^+^ (2), 113 (6), 103 (4), 86 (9), 73 (32), 57(39), 45(73), 41(100); CI (CH_4_), *m*/*z* calcd, 145.09; found, 144.95 [MH]^+^; APCI, HRMS *m*/*z*: calcd, 145.0865; found, 145.0863 [M + H]^+^ for C_7_H_13_O_3_.

### Synthesis of Cyclic Acetal Homopolymers

Homopolymers of cyclic acetals were synthesized according to procedures described previously.^23^ 10 mL of freshly distilled methylene chloride, 90 mg (1 mmol) of butane-1,4-diol (1 mmol of ethylene glycol was used instead of butane-1,4-diol in the case of Cl-DXL and Allyl-DXL), and 44 μL (0.5 mmol) of triflic acid were placed in a Schlenk flask filled with argon. Subsequently, 10 g (98 mmol) of DXP or Cl-DXL (82 mmol) or Allyl-DXL (69 mmol) was added dropwise to the reaction mixture within 8 h, and the polymerization was left for 24 h at room temperature. CaO was added to the reaction mixture to quench the polymerization. Next, the reaction mixture was diluted with 10 mL of dichloromethane, CaO was filtered off, and the polymer was precipitated with cold hexane. The obtained solid was dried under a vacuum until a constant weight was reached. In the case of Cl-DXL and Allyl-DXL, homopolymerization was also conducted at 2 and −15 °C within 192 h. The PDXP polymer was obtained with a yield of 60%.

#### PDXP

^1^H NMR, CDCl_3_, 400 MHz, δ (ppm): 4.70 (2H, s, HOCH_2_CH_2_CH_2_CH_2_OCH_2_O– end group), 4.68 (2H, s, –OCH_2_CH_2_CH_2_CH_2_OCH_2_O−), 3.68 (2H, q, HOCH_2_CH_2_CH_2_CH_2_OCH_2_O– end group), 3.56 (4H, q, –OCH_2_CH_2_CH_2_CH_2_OCH_2_O−), 1.68 (4H, m, –OCH_2_CH_2_CH_2_CH_2_OCH_2_O−), 1.62 (2H, m, HOCH_2_CH_2_CH_2_CH_2_OCH_2_O– end group)

^13^C NMR, CDCl_3_, 100 MHz, δ (ppm): 95.12 (1C, s, –OCH_2_CH_2_CH_2_CH_2_OCH_2_O−), 91.60 (1C, s, HOCH_2_CH_2_ CH_2_CH_2_OCH_2_O– end group), 67.30 (1C, s, –OCH_2_CH_2_CH_2_CH_2_OCH_2_O−), 62.30 (1C, s, HOCH_2_CH_2_CH_2_CH_2_OCH_2_O– end group), 29.70 (1C, s, HOCH_2_CH_2_CH_2_CH_2_OCH_2_O– end group), 26.48 (1C, s, –OCH_2_CH_2_CH_2_CH_2_OCH_2_O−).

### Synthesis of Lactide/DXP Copolymers (PLA/PDXP)

The synthesis of lactide/DXP copolymers was performed according to previously published procedures.^22^ LA (4.5 g, 31.25 mmol) was placed in a Schlenk flask and dried under vacuum for 2 h. Subsequently, the flask was filled with argon and closed with a silicon septum. Ten mL of distilled methylene chloride, 39 μg (0.44 mmol) of butan-1,4-diol, and 38 μL (0.43 mmol) of triflic acid were introduced to the reaction vessel through the septum. To enhance the high contribution of the activated monomer (AM) mechanism and gain control over the copolymer molar mass, polymerization was carried out with a low instantaneous concentration of DXP in the reaction mixture. Thus, after 30 min of lactide polymerization, the appropriate amount of DXP (1.5 3.12, 5.6, and 12.12 mmol) was introduced to the reaction mixture within 8 h. Then, polymerization was performed for 24 or 48 h at 30 °C. Next, purification was performed as conducted for DXP homopolymerization. The polymers were obtained in a yield of 90%.

#### PLA/PDXP

^1^H NMR, CDCl_3_, 400 MHz, δ (ppm): 5.17 (1H, q, –CH(CH_3_)CO−), 4.79 (2H, s, –OCH_2_CH_2_CH_2_CH_2_OCH_2_O–CO–CH(CH_3_)−), 4.40 (1H, q, LA-CH–OH end group), 4.13 (4H, br, –OCH_2_CH_2_CH_2_CH_2_OCH_2_O–CO–CH(CH_3_)−), 1.70 (4H, br, –OCH_2_CH_2_CH_2_CH_2_OCH_2_O–CO–CH(CH_3_)−), 1.54 (3H, d, –CO–CH(CH_3_)−), 1.50–1.43 (3H, m, –CO–CH(CH_3_)–OH).

^13^C NMR, CDCl_3_, 100 MHz, δ (ppm): 175.2 (1C, s, C=O end group), 172.4 (1C, s, –CH_2_CH_2_CH_2_OC(O)CH−), 170.1–169.6 (1C, m, –(CH_3_)CH-C(O)– homosequences), 92.8 (1C, s, –OCH_2_CH_2_CH_2_CH_2_OCH_2_O–CH(CH_3_)−), 70.80 (1C, s, –OCH_2_CH_2_CH_2_CH_2_OCH_2_O–CH(CH_3_)−), 69.4–68.7 (1C, m, –CH(CH_3_)-homosequences), 68.5 (1C, s, –CH(CH_3_)–OH end group), 64.70 (1C, s, –OCH_2_CH_2_CH_2_CH_2_OCH_2_O–CH(CH_3_)−), 25.01 (1C, s, –OCH_2_CH_2_CH_2_CH_2_OCH_2_O–CH(CH_3_)−), 20.41 (1C, s, –CH(CH_3_)–OH end group), 18.36 (1C, s, –OCH_2_CH_2_CH_2_CH_2_OCH_2_O–CH(CH_3_)−), 17.01–16.03 (1C, m, –CH(CH_3_)– homosequences).

### Synthesis of Lactide/Cl-DXL (PLA/PCl-DXL) or Lactide/Allyl-DXL (PLA/PAllyl-DXL) Copolymers

The copolymerization of lactide with substituted cyclic acetals does not require the dropwise addition of acetals to the reaction mixture to avoid the cyclization reaction. To obtain a functionalized polyester, LA (4.5 g, 31.25 mmol) was placed in a Schlenk flask and dried under vacuum for 2 h. Subsequently, the flask was filled with argon and closed with a silicon septum. Ten mL of distilled methylene chloride, 25 μg (0.44 mmol) of ethylene glycol, and 38 μL (0.43 mmol) of triflic acid were introduced to the reaction vessel through the septum. After 30 min of lactide polymerization, the appropriate amount of Cl-DXL or Allyl-DXL (15.6 mmol) was introduced to the reaction mixture, and then, polymerization proceeded for 24 h at room temperature. Then, purification was performed, as in the case of DXP homopolymerization. Copolymerization was also performed at 2 °C (48 h) and −15 °C (72 h). In the case of Cl-DXL, the synthesis was additionally performed on a large scale (25 g of LA). PLA/Cl-DXL was afforded as a white powder, whereas PLA/Ally-DXL was a brownish powder (no possibility of determining the mass from the MALLS detector). The PLA/PCl-DXL copolymers were obtained with a yield of 85%, while PLA/PAllyl-DXL was obtained with a yield of 80%.

#### PLA/PCl-DXL

^1^H NMR, CDCl_3_, 400 MHz, δ (ppm): 5.17 (1H, q, –CH(CH_3_)CO−), 4.82 (2H, s, –CO–CH(CH_3_)OCH_2_OCH_2_CH(CH_2_Cl)O−), 4.47–4.42 (3H, m, LA-CH–OH end group, –CO–CH(CH_3_)OCH_2_OCH_2_CH(CH_2_Cl)O−), –CH(CH_3_)OCH_2_OCH_2_CH(CH_2_Cl)O−), 3.70–3.57 (4H, m, –CH(CH_3_)OCH_2_OCH_2_CH(CH_2_Cl)O−), 1.76 (3H, d, –CH(CH_3_)O−), 1.54–1.44 (3H, m, –CH(CH_3_)OH, –CH(CH_3_)OCH_2_OCH_2_CH(CH_2_Cl)O−).

^13^C NMR, CDCl_3_, 100 MHz, δ (ppm): 175.2 (1C, s, C=O end group), 172.1 (1C, s, –COCH(CH_3_)OCH_2_OCH_2_CH(CH_2_Cl)O−), 169.9–169.7 (1C, m, –(CH_3_)CHC(O)– homosequences), 92.8 (1C, s, –COCH(CH_3_)OCH_2_OCH_2_CH(CH_2_Cl)O−), 71.80 (1C, s, COCH(CH_3_)OCH_2_OCH_2_CH(CH_2_Cl)O−), 69.4–68.7 (1C, m, –CH(CH_3_)-homosequences), 66.5 (1C, s, –CH(CH_3_)–OH end group), 62.8 (1C, s, –COCH(CH_3_)OCH_2_OCH_2_CH(CH_2_Cl)O−), 41.14 (1C, s, –COCH(CH_3_)OCH_2_OCH_2_CH(CH_2_Cl)O−), 20.47 (1C, s, –CH(CH_3_)–OH end group), 18.42 (1C, s, –COCH(CH_3_)OCH_2_OCH_2_CH(CH_2_Cl)O−), 16.08 (1C, s, –CH(CH_3_)– homosequences).

#### PLA/PAllyl-DXL

^1^H NMR, CDCl_3_, 400 MHz, δ (ppm): 5.90–5.73 (1H, m, CH_2_=CH–CH_2_–O−), 5.30–5.10 (3H, m, CH_2_=CH–CH_2_–O–, –CH(CH_3_)CO−), 4.81 (2H, s, –COCH(CH_3_)OCH_2_OCH_2_CH(CH_2_O−)O−), 4.43 (1H, s, LA-CH–OH end group), 4.34 (3H, overlapped –COCH(CH_3_)OCH_2_OCH_2_CH(CH_2_O−)O−) 3.99 (2H, m, CH_2_=CH–CH_2_–O−), 3.54 (2H, m, –COCH(CH_3_)OCH_2_OCH_2_CH(CH_2_O−)O−), 1.61 (3H, d, –CH(CH_3_)O−), 1.54–1.44 (6H, m, –CH(CH_3_)OH, –COCH(CH_3_)OCH_2_OCH_2_CH(CH_2_O−)O−).

^13^C NMR, CDCl_3_, 100 MHz, δ (ppm): 175.2 (1C, s, C=O end group), 172.1 (1C, s, –COCH(CH_3_)OCH_2_OCH_2_CH(CH_2_O−)O−), 169.9–169.7 (1C, m, –(CH_3_)CHC(O)– homosequences), 133.88 (1C, s, CH_2_=CH–CH_2_–O−), 117.52 (1C, s, CH_2_=CH–CH_2_–O−), 92.82 (1C, s, –COCH(CH_3_)OCH_2_OCH_2_CH(CH_2_O−)O−), 72.27 (1C, d, CH_2_=CH–CH_2_–O−), 70.66 (1C, s, –COCH(CH_3_)OCH_2_OCH_2_CH(CH_2_O−)O−), 69.4–68.7 (1C, m, –CH(CH_3_)-homosequences), 67.80 (1C, d, –COCH(CH_3_)OCH_2_OCH_2_CH(CH_2_O−)O−), 63.55 (1C, d, –COCH(CH_3_)OCH_2_OCH_2_CH(CH_2_O−)O−), 20.39 (1C, s, –CH(CH_3_)OH), 18.28 (1C, s, –COCH(CH_3_)OCH_2_OCH_2_CH(CH_2_O−)O−), 16.54 (1C, s, –CH(CH_3_)O−).

### Modification of PLA/PCl-DXL Copolymers by Azide and Alkyne Compounds

The reaction of PLA/PCl-DXL with sodium azide was performed to show the possibility of the copolymer being functionalized. Two g of PLA/PCl-DXL containing 13 mol % chloromethyl groups and 300 mg (4.28 mmol) of sodium azide were dissolved in 10 mL of distilled DMF. Then, the reaction was filled with argon and maintained at 55 °C for 24 h. After that, the solid was centrifuged, and DMF was evaporated with a rotary evaporator. Subsequently, the polymer was dissolved in methylene chloride and the rest of the organic salt was filtered off. The polymer was precipitated into diethyl ether. The conversion of the chloromethylene group to the azide group was checked by ^13^C NMR. After successful conversion of the chloromethyl group into azide one, the copolymer was reacted with propargyl alcohol and propargyl glycidyl ether as follows: 200 mg of copolymers containing 13 mol % azide groups, 0.44 mmol alkyne compounds (propargyl alcohol or propargyl glycidyl ether), 0.24 mmol CuBr was dissolved in 2 mL of THF. After that, 0.24 mmol pentamethyldiethylenetriamine (PMDTA) was added to the reaction mixture and was stirred for 24 h at 40 °C. Then, the reaction was passed through the silica gel column and precipitated to the hexane/ether (1:1) solution. The obtained products were dried under vacuum to a constant weight. The PLA/PCl-DXL copolymers were obtained with a yield of 92%.

^13^C NMR, CDCl_3_, 100 MHz, δ (ppm): 175.2 (1C, s, C=O end group), 172.1 (1C, s, –COCH(CH_3_)OCH_2_OCH_2_CH(CH_2_Cl)O−), 169.9–169.7 (1C, m, –(CH_3_)CHC(O)– homosequences), 92.8 (1C, s, COCH(CH_3_)OCH_2_OCH_2_CH(CH_2_Cl)O−), 71.80 (1C, s, COCH(CH_3_)OCH_2_OCH_2_CH(CH_2_Cl)O−), 69.4–68.7 (1C, m, –CH(CH_3_)-homosequences), 66.5 (1C, s, –CH(CH_3_)–OH end group), 62.8 (1C, s, COCH(CH_3_)OCH_2_OCH_2_CH(CH_2_Cl)O−), 51.00 (1C, s, COCH(CH_3_)OCH_2_OCH2CH(CH_2_N_3_)O−), 20.47 (1C, s, –CH(CH_3_)–OH end group), 18.42 (1C, s, COCH(CH_3_)OCH_2_OCH_2_CH(CH_2_Cl)O−), 16.08 (1C, s, –CH(CH_3_)– homosequences).

### Modification of PLA/PAllyl-DXL Copolymers by Click Reaction

The reaction of PLA/PAllyl-DXL with propanethiol, mercaptoethanol, or thioglycolic acid was performed to show the possibility of the copolymer being functionalized. Briefly, 1 g of PLA/PAllyl-DXL containing 10 mol % of a double bond, 3.9 mmol of appropriate thiol, 20 mg (0.12 mmol) of AIBN, and 10 mL of THF was placed in a Schlenk tube. The reaction mixture was degassed in an ultrasound bath for 30 min. Then, the Schlenk tube was placed in an oil bath at 60 °C for 24 h. After that, the reaction mixture was cooled, precipitated to cold diethyl ether, and dried under a vacuum. The disappearance of the signal belonging to the double bond was checked by ^1^H NMR. The PLA/PAllyl-DXLopolymers were obtained with a yield of 82%.

^1^H NMR, CDCl_3_, 400 MHz, δ (ppm): 5.15 (1H, q, –CH(CH_3_)CO−), 4.81 (2H, s, –COCH(CH_3_)OCH_2_OCH_2_CH(CH_2_O−)O−), 4.43 overlapped –COCH(CH_3_)OCH_2_OCH_2_CH(CH_2_O−)O−), 4.34 (1H, s, LA-CH–OH end group), 3.54 (4H, m, –COCH(CH_3_)OCH_2_OCH_2_CH(CH_2_O−)O–, CH_3_–CH_2_–CH_2_–S–CH_2_–CH_2_–CH_2_–O−), 2.55 (2H, m, CH_3_–CH_2_–CH_2_–S–CH_2_–CH_2_–CH_2_–O−), 2.49 (2H, m, CH_3_–CH_2_–CH_2_–S–CH_2_–CH_2_–CH_2_–O−), 1.61 (5H, d, –CH(CH_3_)O–, CH_3_–CH_2_–CH_2_–S–CH_2_–CH_2_–CH_2_–O−), 1.54–1.44 (6H, m, –CH(CH_3_)OH, –COCH(CH_3_)OCH_2_OCH_2_CH(CH_2_O−)O−), 1.05 (3H, t, CH_3_–CH_2_–CH_2_–S–CH_2_–CH_2_–CH_2_–O−).

### Acid Triggered Degradation of PLA/PDXP_5_ and PLA/PCl-DXL Copolymers

The hydrolytic degradation assay of the purified copolymers was performed according to a previously published method.^22^ For example, 10 mg of PLA/PDXP_10_ or PLA/PCl-DXL_10_ was placed in the NMR tube and dissolved in 680 μL of detreated acetonitrile (CD_3_CN), 10 μL of concentrated HCl solution (35%) was added to the NMR tube, and the spectra were recorded after selected time interval within 15 h at 40 °C. For the analyzed copolymers, the molar ratio of acid to acetal groups was kept constant (1:1).

### Characterization

^1^H NMR, ^13^C NMR, and 2D HSQC NMR spectra were recorded on an Avance Neo 400 NMR spectrometer. ^1^H DOSY NMR analysis and degradation assay were performed on a Bruker DRX500 spectrometer operating at 500 MHz (11.7 T). The molar masses (*M*_n_) of copolymers and polydispersity were determined by size-exclusion chromatography using an Agilent Pump 1100 Series instrument equipped with two PLGel 5 μm MIXED-C columns. A Wyatt Optilab Rex differential refractometer (RI) and a Dawn Eos8 (Wyatt Technology Corporation) laser photometer were used as detectors. Dichloromethane was used as an eluent (0.8 mL/min, 25 °C). Gas chromatography was analyzed using a Shimadzu QP2010 Plus apparatus with a Zebron ZB-5MSi Capillary GC column (30 m × 0.25 mm  ×  0.25 μm) connected with the quadrupole mass spectrometer, Shimadzu QP2010 Ultra, with electron impact (EI, 70 eV) and chemical positive ionization (PCI, CH_4_). The carrier gas was helium at 0.97 mL/min. The injector and ion source temperatures were set at 250 and 200 °C, respectively. The temperature program was as follows: hold at 35 °C for 5 min, heating to 240 °C at a rate of 10 °C/min, and hold at 240 °C for 5 min. The MS was scanned from 35 to 600 amu (atomic mass unit) by selecting full-scan mode. Additionally, Allyl-DXL mass spectrometry detection using an APCI source in positive high-resolution mode was performed. To ensure accurate mass measurements, the data were collected in centroid mode. Weight was corrected using leucine enkephalin solution as an external reference (Lock-SprayTM). The optimized source parameters included a corona voltage of 0.5 kV, probe temperature 400 °C, source temperature 80 °C, desolvation gas (nitrogen) flow rate 500 L/h, nebulizer gas pressure 6.5 bar. Mass spectra were recorded in the *m*/*z* range from 50 to 1200 Da. Differential scanning calorimetry (DSC) analyses were performed under a nitrogen atmosphere at heating and cooling rates of 10 °C min^–1^ on a DSC 2500 Discovery system (TA Instruments). The measurements were performed at temperatures ranging from −90 to 200 °C for homopolymers and copolymers. The temperature and heat flows were both calibrated with indium. Thermogravimetric analysis (TGA) was performed under nitrogen flow by heating the samples from ambient temperature to 500 °C at a heating rate of 20 °C min^–1^ on a Hi-Res TGA 2950 thermogravimetric analyzer (TA Instruments). The measurements were conducted by placing ∼5 mg of polymer samples in a measuring cell.

## Results and Discussion

### Synthesis of the Functionalized Cyclic Acetals

In the strategy we proposed, it is essential to use appropriate comonomers that can introduce the necessary functional groups to the polyester chain and simultaneously influence or improve the material degradation properties. For this purpose, we designed two cyclic acetals that differ in their pendant functionality. Cyclic acetals may be prepared by condensation of formaldehyde or higher aldehydes with diols in the presence of an acid catalyst.^24^ Thus, in our work, in the first step, 3-allyloxypropane-1,2-diol and 3-chloropropane-1,2-diol were selected for the reaction with paraformaldehyde to afford the formation of five-membered cyclic acetals fitted with allyl- or chloro-functional groups (structures shown in Scheme S1). Additionally, for comparative studies, a nonfunctionalized seven-membered acetal was prepared using butane-1,4-diol. We observed that reactions leading to the formation of substituted cyclic acetals proceeded with high yields (80 and 85%) and were close to the reaction yield for the unsubstituted acetal, which was almost 90%. Notably, the synthesis methodology allowed for the reaction with diols having a chlorine substituent as well as an unsaturated moiety, and the applied conditions (110 °C, toluene, pTSA) prevented functional group side reactions during acetal formation.

The required structures of the prepared monomers were confirmed based on the ^1^H and ^13^C NMR spectra (Figures S1 and S2). The characteristic signals belonging to the acetal bond (−OCH_2_O−) are observed in the range of 4.80–5.00 ppm and 95.60–94.35 for the proton and carbon, respectively. Additionally, the ^1^H NMR spectra demonstrated a lack of a free aldehyde peak at 9.60 ppm or free alcohols.

Functionalized and nonfunctionalized cyclic acetals were purified by distillation to remove the acidic catalyst, unreacted diols, toluene, or other side products. The high purity of the synthesized cyclic monomers was confirmed by GC/MS analysis. In all cases, only one well-defined peak belonging to the particular cyclic acetal was observed in the GC chromatogram (Figure S3a). No additional peaks from volatile compounds, such as diols or acyclic acetals, were detected. In the MS spectra (EI ionization), weak molecular peaks at 121, 102, and 143 *m*/*z* were detected for Cl-DXL, DXP, and Allyl-DXL, respectively (Figure S3b–d). The mass spectrum of DXP shows a weak ion peak at the parent mass-to-charge ratio, *m*/*z* 102, of C_5_H_10_O_2_^+^ (Figure S3c). Fragment peaks clustered at *m*/*z* 71 and 42 are characteristic of ions C_4_H_7_O^+^ and C_3_H_6_^+^, respectively. The primary process is the loss of CH_2_O.^25^ In the mass spectra of 4-substituted 1,3-dioxolanes, the characteristic feature is the loss of H;^26^ therefore, for Cl-DXL and Allyl-DXL, we observed weak parent ion signals with *m*/*z* 121 and 142, respectively (Figure S3b, d). Additionally, for Cl-DXL, a typical chlorine isotope distribution in the molecular peak was observed (Figure S3b) in the spectrum.^27^ GC/MS and ^1^H NMR spectra showed that two functional five-membered acetals and one nonfunctional seven-membered acetal were successfully synthesized. These results demonstrated that the reaction with paraformaldehyde with a proper diol is a simple and straightforward way to achieve functionalized cyclic acetals.

### Synthesis of Functionalized Polylactides via Copolymerization with Cyclic Acetals

The main goal of this work was to develop functional, degradable lactide copolymers that can be prepared under metal-free conditions. In accordance with the above assumption, we designed the cationic copolymerization of lactide (LA) with functionalized cyclic acetals under conditions that had been successfully applied previously for the copolymerization of lactide with 1,3-dioxolane.^22^ Before investigating the copolymerization, we tested the homopolymerization abilities of Allyl-DXL and Cl-DXL (Table 1). The homopolymerization of DXP was already reported by Pan et al.^28^ It was shown that the cationic polymerization of DXP initiated by triflic acid in the presence of diols proceeded according to the Activated Monomer (AM) mechanism, at a high mole ratio of diol/initiator in which monomer was slowly added into the polymerization system.

**Table 1:** Cationic Ring-Opening Homopolymerization of Cyclic Acetals and Their Copolymerization with LA^a^

				reactive mixture composition [mol %]			conversion [%]^b^		*M*_n_*SEC*^c^ [g/mol]			copolymer composition [mol %]^d^		average number of repeating units^f^		
runs	sample	*T* (°C)	time [h]	LA	acetal	theoretical molar mass [g/mol]	LA	acetal	RI	MALS	*D̵*^c^	LA	acetal	LA	acetal	homo LA block/acetal^g^
1	PDXP	30	24	0	100	10 000	0	87	12 000	9000	1.7	0	100	0	83.0	
2	PLA/PDXP_5_	30	24	97	4.5	10 500	95	98	9500	12 500	1.8	95	5	62.0	4.6	14
3	PLA/PDXP_10_	30	48	93	9.0	11 000	94	95	10 000	12 500	2.2	89	10	62.0	10.0	6.4
4	PLA/PDXP_18_	30	48	84	15.0	11 000	96	95	10 400	13 000	2.2	84	18	60.0	16.0	4.3
5	PLA/PDXP_26_	30	48	72	27.0	11 000	98	94	11 000	9000	1.9	73	26	55.0	28.0	1.9
6	PLA/PDXP_11_	30	48	91	9.1	25 000	99	98	16 000	17 000	1.7	88	11	98.0	17.0	6.3
7	PCl-DXL	30	192	0	100	11 800	100	0	0	0	0	0	0	0	0	
8	PCl-DXL	2	192	0	100	11 800	100	0	0	0	0	0	0	0	0	
9	PCl-DXL	–15	192	0	100	11 800	100	0	0	0	0	0	0	0	0	
10	PLA/PCl-DXL_10_	30	24	67	33.3	11 000	97	20	13 000	14 000	1.9	90	10	81.0	13.0	8.0
11	PLA/PCl-DXL_16_	2	48	67	33.3	11 000	90	30	11 000	13 000	1.9	82	16	65.0	17.0	4.5
12	PLA/PCl-DXL_22_	–15	72	67	33.3	11 000	50	40	11 500	13 500	2.1	78	22	62.0	21.0	3.8
13	PLA/PCl-DXL_9_	30	24	67	33.3	25 000	96	21	17 500	17 000	1.8	91	9	110.0	13.0	8.1
14	PAllyl-DXL	30	48	0	0	14 000	100	0	0	0	0	0	0	0	0	
15	PAllyl-DXL	2	192	0	0	14 000	100	0	0	0	0	0	0	0	0	
16	PAllyl-DXL	–15	192	0	0	14 000	100	0	0	0	0	0	0	0	0	
17	PLA/PAllyl-DXL_10_	30	24	66.7	33.3	11 000	94	20	10 000	nd^e^	1.4	90	10	62.5	7.0	9.0
18	PLA/PAllyl-DXL_14_	2	48	67	33.3	11 000	91	32	10 500	nd^e^	1.7	86	14	62.5	10.0	6.3
19	PLA/PAllyl-DXL_22_	–15	72	67	33.3	11 000	55	40	9500	nd^e^	1.7	82	22	54.0	12.0	2.7
20	PLA/PAllyl-DXL_9_	30	24	67	33.3	25 000	92	22	19 000	nd^e^	2.0	91	9	120.0	12.0	10.0

a Copolymerization conditions: for PDXP/PLA triflic acid (0.43 mmol) as catalyst, butylene glycol (0.44 mmol) as initiator; for PLA/PCl-DXL and PLA/PAllyl-DXL triflic acid (0.43 mmol) and ethylene glycol (0.44 mmol) as initiator.

b Comonomer conversion determined from the NMR spectra.

c The number-average molar masses were estimated for purified homo or copolymers by size-exclusion chromatography (SEC) with a MALLS detector (d*_n_*/d*_c_*_DXP_ = 0.030, d*_n_*/d*_c_*_CDXL_ = 0.044) or an RI detector calibrated with polystyrene standards. *D̵* = *M*_w_/*M*_n_ was measured by SEC with an RI detector.

d The composition of the copolymer was calculated from the ^1^H NMR spectra.

e In the case of the PLA/Allyl-DXL copolymers, the molar mass was only estimated from the RI detector (polystyrene calibration). The molar mass from the MALLS detector was not measured because of the brownish color of the purified copolymers.

f The average number of repeating units in the copolymer was calculated taking into account its molar mass (RI detection), composition (molar content of the given comonomer), and molar mass of the repeating unit according to the following formula: *M*_ncopolymer_ × [comonomer mol %]/*M*_comonomer_.

g Calculated from ^1^H NMR spectra taking into account the signal from the −CH– group (LA–LA) and signal belonging to the –OCH_2_O– acetal group.

**Figure 1:**
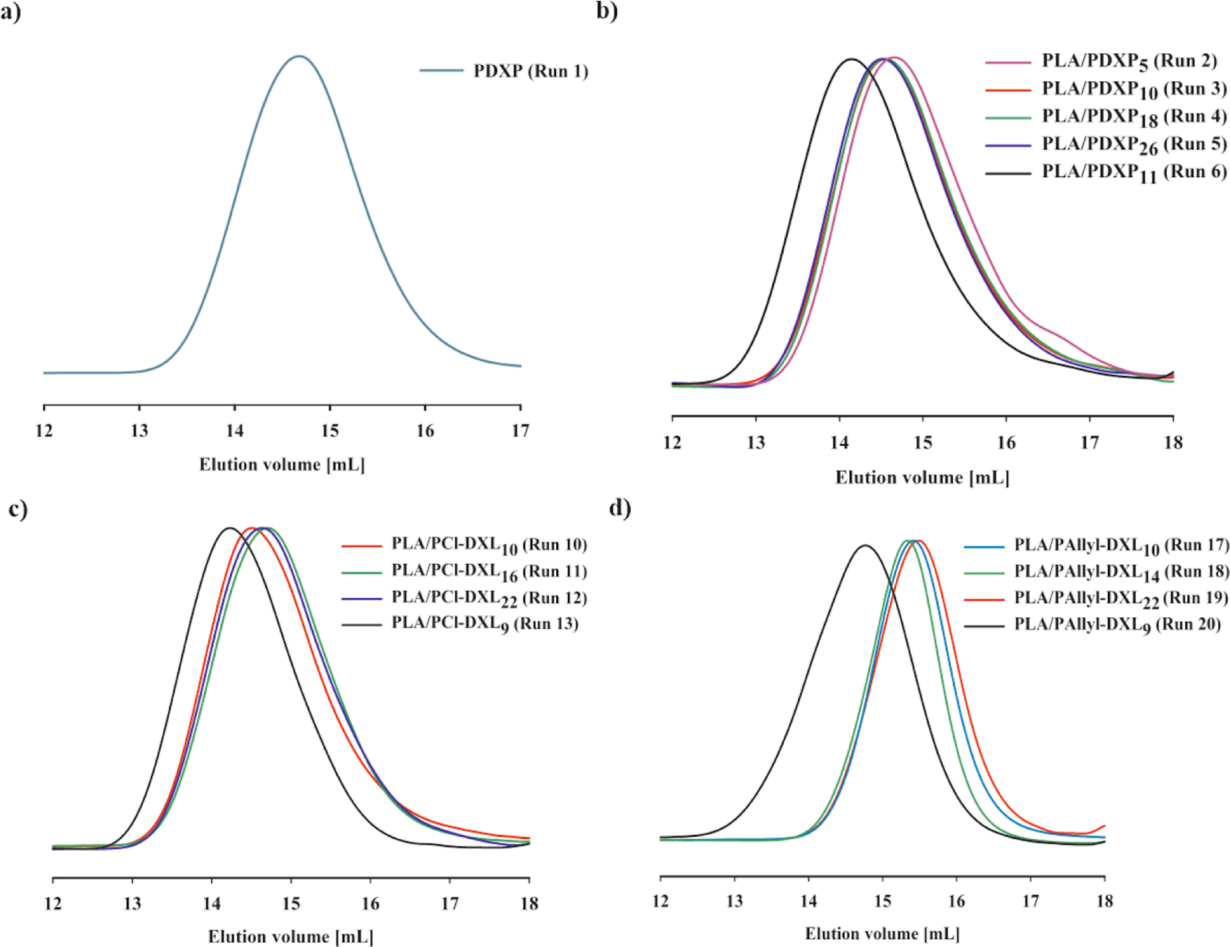
GPC traces of prepared (a) PDXP homopolymer, (b) PLA/PDXP, (c) PLA/PCl-DXL, and (d) PLA/PAllyl-DXL copolymers.

Thus, for DXP applying butane-1,4-diol as an initiator and triflic acid as a catalyst after 24 h at 30 °C, we easily obtained a homopolymer (PDXP) with *M*_n_ = 12 000 g/mol and *M*_w_/*M*_n_ = 1.7 (Figure 1 and Table 1, run 1). The ^1^H NMR showed typical chemical shifts at 4.68, 3.56, and 1.68 ppm for –OCH_2_OCH_2_CH_2_CH_2_CH_2_O–, –OCH_2_OCH_2_CH_2_CH_2_CH_2_O–, and –OCH_2_OCH_2_CH_2_CH_2_CH_2_O–, respectively. The ^1^H, ^13^C, and the structure of PDXP are shown in Figure S4a,b, whereas the SEC chromatogram is presented in Figure 1a.

However, using the same conditions for Cl-DXL or Allyl-DXL polymerization, we did not obtain the corresponding homopolymers (Table 1, runs 7 and 14 for PCl-DXL and PAllyl-DXL, respectively). This observation correlates with previously reported results regarding the homopolymerization of substituted acetals in which substitution causes lower negative enthalpy.^29^ To undergo a significant extent of ring-opening polymerization, cyclic monomers must exhibit a negative free energy of polymerization under given conditions. In general, substituents invariably make Δ*G* less negative and are liable to change the sign of Δ*G* from negative to positive when five-, six-, and seven-membered rings are substituted.^30^

It is well-known that the polymerization of cyclic acetals characterizes the low ceiling temperature;^31^ therefore, typically decreasing the temperature (thus decreasing the −*T*Δ*S* factor) should influence the thermodynamics of the polymerization process and contribute to the formation of the polymer. For example, it was reported that to prepare poly(4-ethyl-1,3-dioxolane), the temperature must be decreased to −20 °C to synthesize oligomers with a molecular weight of approximately 400 g/mol.^32^ Therefore, we examined the effect of the temperature on the course of Allyl-DXL and Cl-DXL homopolymerization carrying out the reactions at 2 and −15 °C (Table 1, runs 8, 9 and 15, 16 for PCl-DXL and PAllyl-DXL, respectively). Nevertheless, neither lowering the temperature nor increasing the reaction time to 192 h enabled the formation of Allyl-DXL or Cl-DXL homopolymers.

The inability of Cl-DXL and Allyl-DXL to form high molar mass homopolymers does not imply that these monomers could not be copolymerized with other suitable comonomers. In principle, copolymerization of nonpolymerizable monomer M_2_ with polymerizable monomer M_1_ may occur up to a maxim um possible incorporation of 50% M_2_ units, corresponding to an alternating copolymer.^30^ For example, γ-butyrolactone, which is often considered “hardly homopolymerizable” at standard temperature and pressure,^33,34^ was successfully copolymerized with some other lactones under these conditions.^35^ Also, Okada et al. showed that Cl-DXL did not form homopolymers but it can be introduced to the polymer chain by copolymerization with 1,3-dioxolane.^36^

**Scheme 1:**
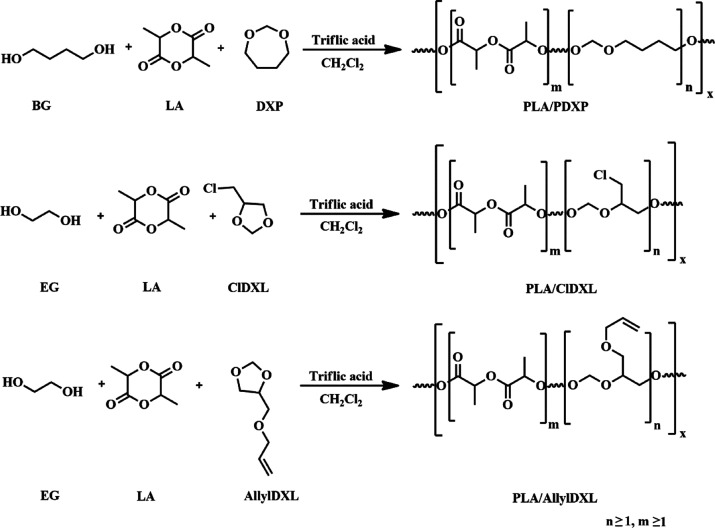
Synthesis of Lactide/Cyclic Acetal Copolymers [Copolymerization Conditions: For PDXP/PLA Triflic Acid (0.55 mmol) as the Catalyst, Butylene Glycol (1 mmol) as the Initiator; for PLA/PCl-DXL and PLA/PAllyl-DXL Triflic Acid (0.43 mmol) and Ethylene Glycol (0.44 mmol) as the Initiator]

Therefore, we tested the ability of the obtained functional acetals to copolymerize with lactide to obtain copolyesters containing functional acetal units. The synthesis is illustrated in Scheme 1.

**Figure 2:**
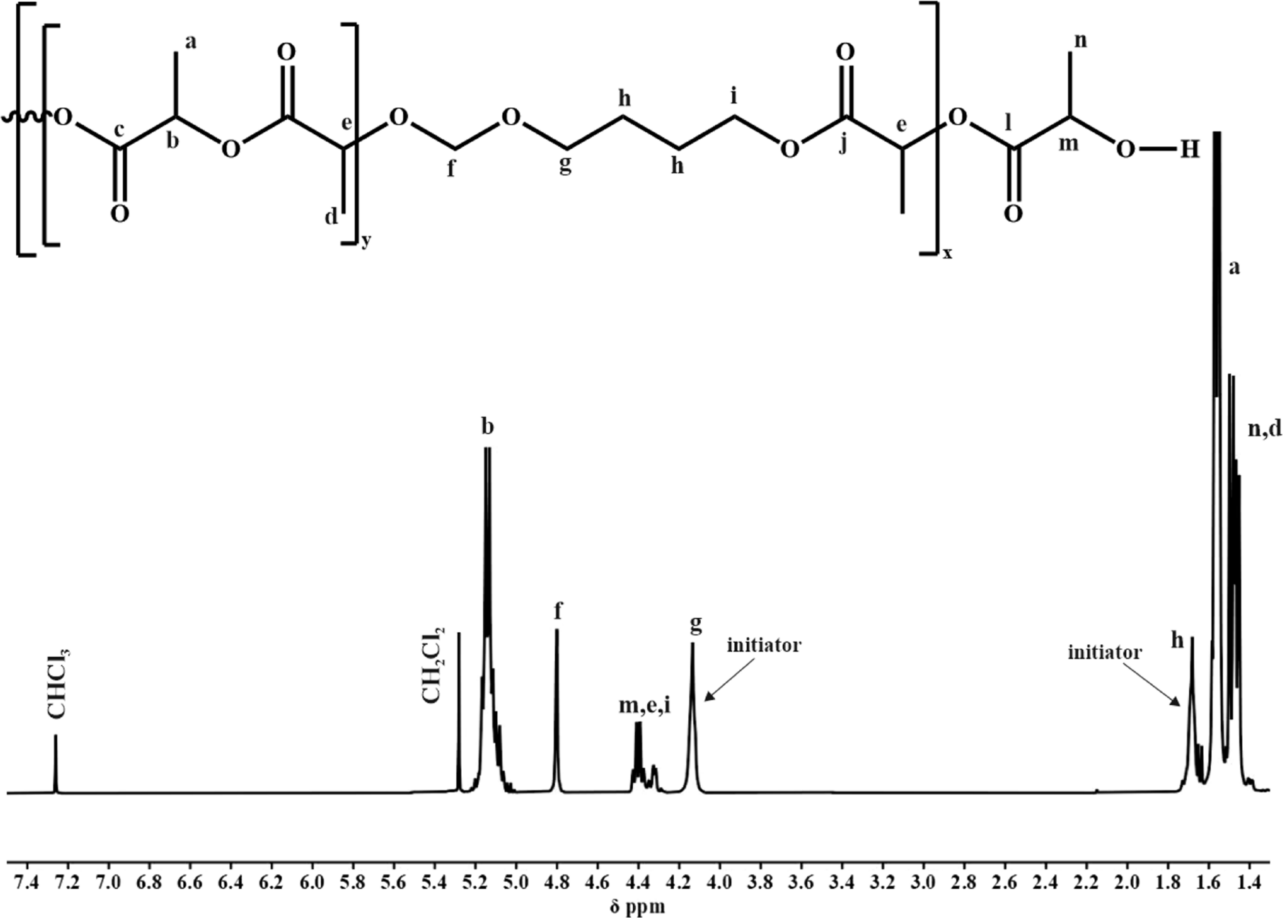
^1^H spectra of PLA/PDXP_5_ (CDCl_3_, 400 MHz).

Nonfunctionalized seven-membered 1,3-dioxepane was also used for the copolymerization, which made it possible to study the effect of the substituent on the cyclic acetal reactivity (Table 1, runs 2–6). As DXP is known to undergo side reactions during homopolymerization that lead to cyclic structure formation,^37^ to shift the mechanism of propagation toward the activated monomer^22^ mechanism and prevent the presence of cycles, the monomer was manually added dropwise within 8 h to the reaction mixture containing lactide, butane-1,4-diol, and triflic acid. As presented in Table 1, high conversion of both comonomers (95–97%) was observed under the applied conditions, and using different contents of DXP in the feed (from 5 to 26 mol %) enabled the preparation of lactide copolymers comprising various acetal units per chain with high yields. The percentage of DXP units incorporated in the copolymer increased with the increase in the content of this comonomer in the polymerization feed. In our work, we used relatively small amounts of functional acetal to largely retain the properties of the unmodified LA homopolymer. SEC chromatograms showed for PLA/PDXP copolymers the unimodal molar masses distribution in the range of 9 000 to 17 000 g/mol (Figure 1b). The copolymers were purified as described in the Experimental Section, and the composition was carefully determined using ^1^H and ^13^C NMR. As we showed previously^22^ for the unsubstituted 1,3-dioxolane, the units are incorporated into the copolymer as –OCH_2_OCH_2_CH_2_CH_2_CH_2_O– not as –OCH_2_CH_2_CH_2_CH_2_OCH_2_O–. In the NMR spectra of the purified product, in addition to the typical polylactide signals (5.17, 4.35, and 1.56 ppm), peaks belonging to the incorporated DXP units were identified. The presence of the acetal–ester structure was confirmed by the peaks described for DXP shifting toward higher frequencies to 4.80, 4.13, and 1.70 ppm (signals f, g, and h in Figure 2, respectively) compared to the chemical shifts observed for the homopolymer.

**Figure 3:**
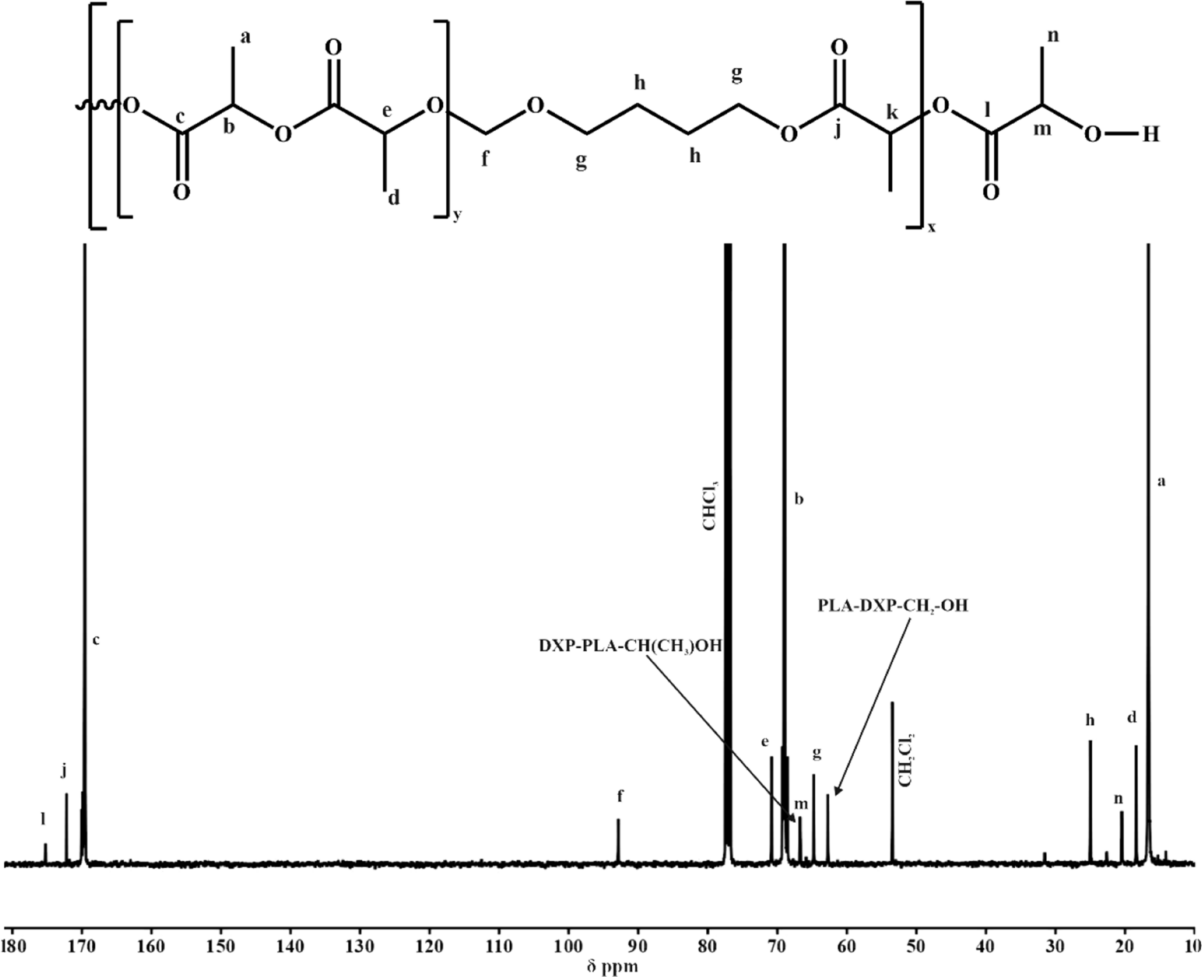
^13^C NMR spectra of the PLA/PDXP_5_ copolymer (CDCl_3_, 100 MHz).

In the ^13^C NMR spectra, apart from signals typical for lactide sequences, signals from the DXP units were also present with chemical shifts that were different from the signals observed in the homopolymer. Additionally, in the carbonyl region, we observed a characteristic signal (172.40, signal j) belonging to LA units directly connected to the DXP moieties in the formed acetal ester unit (Figure 3). To support the observation obtained from ^1^H and ^13^C NMR, 2D HSQC NMR was employed to estimate the correlation between carbon and the directly attached protons (Figure S5). All these analyses proved that the PLA/PDXP copolymer was formed successfully.

The copolymerization of lactide with Cl-DXL and Allyl-DXL does not require the dropwise addition of substituted acetals to the reaction mixture to avoid the cyclization reaction. Due to the inability of these monomers to homopropagation, the formation of longer homosequences is hindered, which limits the formation of undesirable cyclic products. Therefore, for both comonomers, the constant content of cyclic acetal (33 mol %) in the feed was kept during copolymerization with lactide (see Table 1, runs 10–13 and runs 17–20 for PLA/PCl-DXL and PLA/PAllyl-DXL, respectively). Different temperatures ranging from −15 to 30 °C were applied to study their effect on the copolymer composition and number of acetal units incorporated into the copolymer chain.

The copolymerization performed at 30 °C within 24 h proceeded with a high lactide conversion (runs 10 and 17); however, the conversion of both cyclic acetals was rather low (approaching 20%), and after purification, the content of acetal units in the copolymer (calculated from ^1^H NMR spectra) was approximately 12 mol %. Decreasing the temperature to 2 °C slowed the reaction (runs 11 and 18), and a 90% conversion of lactide was reached after 48 h. Additionally, only a small increase in the conversion of cyclic acetals to 30 and 32% was observed for Cl-DXL and Allyl-DXL, respectively. For −15 °C, the acetal conversion reached 40%, and the amount of acetal incorporated did not exceed 22% (runs 12 and 19). However, under this condition, the polymerization of lactide significantly slowed down, and after 72 h, the conversion reached only 50%. Therefore, copolymerization with acetals was not performed below −15 °C because of a low cyclic ester conversion.

Size-exclusion chromatography showed that the elution peaks of the obtained copolymers were symmetrical and uniform (*D̵* = 1.4–2.1), supporting the formation of the desired copolymers (Figure 1c,d). As presented in Table 1, the molar masses of the synthesized functional copolyesters after purification are in the range from 10 000 to 19 000 g/mol. Runs 6, 13, and 20 represent copolymers with the highest molecular weight achievable by using the current experimental procedures. Further attempts to obtain copolymers with *M*_n_ = 50 000 g/mol, by increasing the ratio of comonomers to the initiator, did not lead to the intended goal.

The conversion of comonomers over time in the cationic copolymerization of lactides with Cl-DXL was investigated. The resulting plots are presented in Figure S6. As described in the Experimental Section, a cyclic acetal was introduced into the polymerization mixture after 30 min of lactide homopolymerization, when the conversion of cyclic ester reached 10%. The measurements confirmed a significantly faster polymerization rate for lactide compared to Cl-DXL. After 550 min, almost complete conversion of lactide (90%) was achieved, while the conversion of Cl-DXL reached 33% at the final stage of polymerization. Judging from the plot, during incorporation into the polymer chain, both comonomers are consumed uniformly which should lead to the formation of copolyesters with a relatively homogeneous structure. Since the substituted acetal in this copolymerization system does not have the ability to propagate, no Cl-DXL/Cl-DXL homodiads will be present in the copolymer. Detailed investigations aimed at determining the microstructure of the copolymer (i.e., the distribution of repeating comonomer units in the chain) are planned as an independent aspect of future research. These studies will aim to elucidate the impact of microstructure on the susceptibility to degradation.

**Scheme 2:**
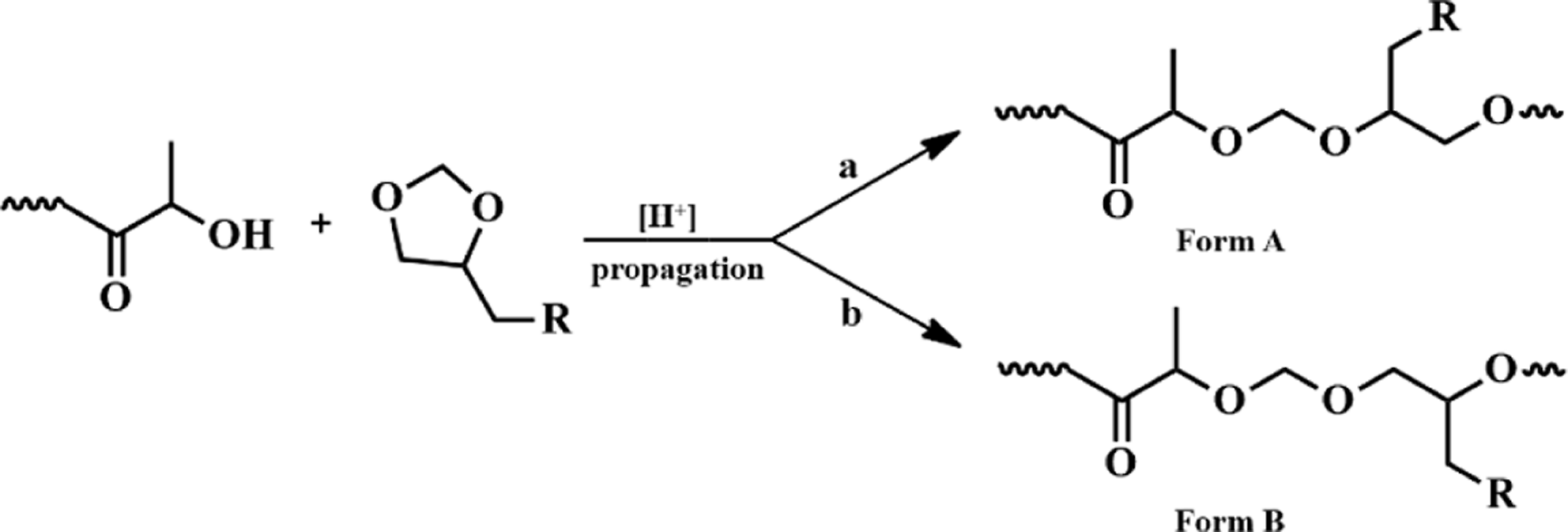
Possible Way of Opening the Acetal Ring during Copolymerization (R–Cl or –OCH_2_CH=CH_2_)

The composition of isolated copolyesters containing units introduced by functionalized cyclic acetals was estimated by ^1^H NMR. The structure analysis is more complicated for PLA/PCl-DXL and PLA/PAllyl-DXL copolymers than for PLA/PDXP copolymers because there are two possible ways of ring opening in the cyclic acetal substituted at the fourth position. This effect was also reported previously by Okada^32^ for unsymmetrical 4-ethyl-1,3-dioxolane and 4-chloromethyl-1,3-dioxolane,^36^ in which the presence of asymmetric carbons led to the formation of a nonregular structure of the polymer chain. Similarly, in our work, functionalized acetals can be introduced into the copolyester chain in two different ways, as shown in Scheme 2.

**Figure 4:**
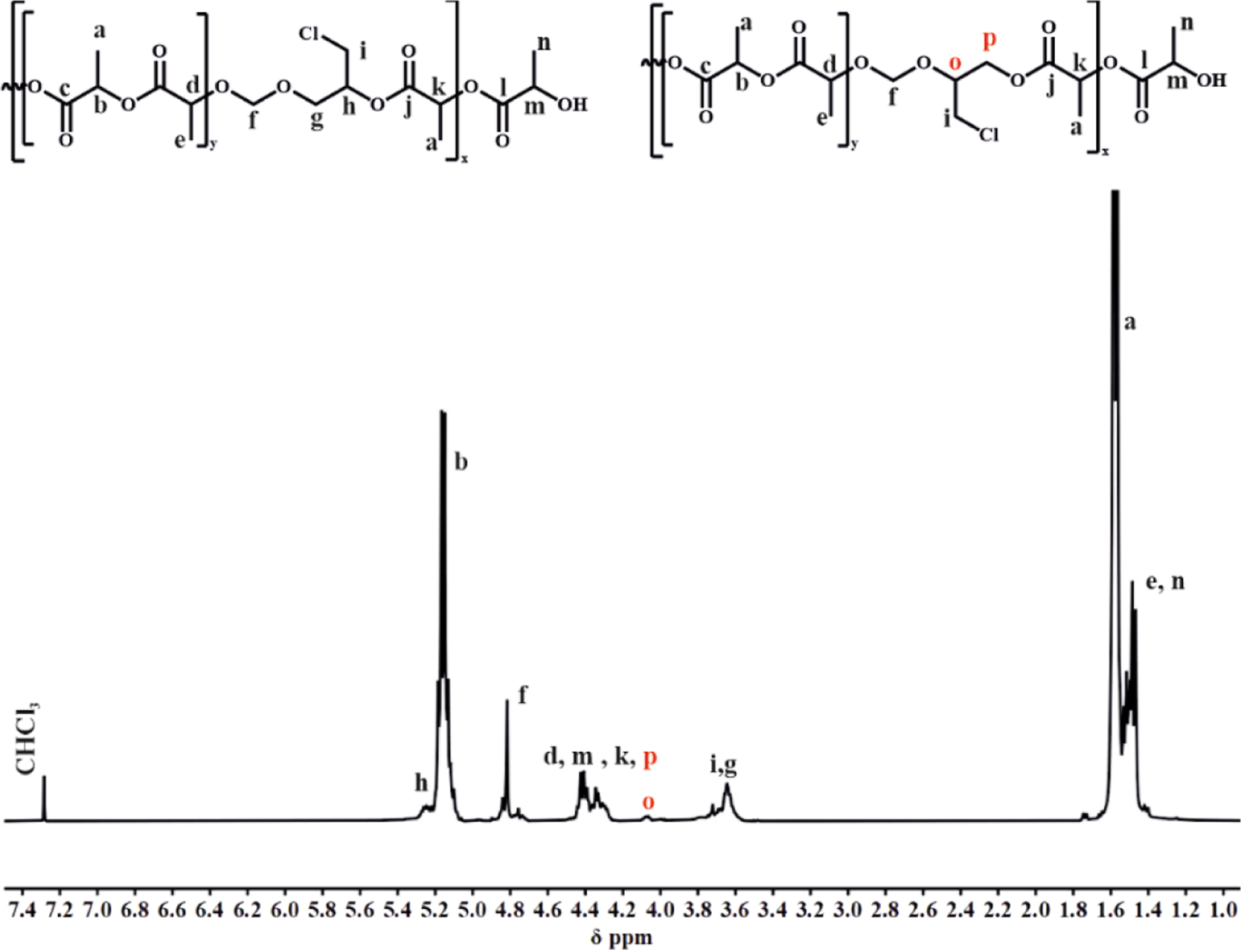
^1^H spectra of the PLA/PCl-DXL copolymer with 12 mol % of Cl-DXL (CDCl_3_, 400 MHz).

In the typical spectrum of the PLA/PCl-DXL copolymer, major peaks from the polyester backbone are observed, together with signals typical for the introduced acetal units. Thus, oxymethylene protons at the acetal-ester unit were identified at 4.80 ppm (signal f). The peaks with chemical shifts in the range from 3.70 to 3.50 ppm (signal g) and 5.20 ppm (signal h) were assigned to the acetal units only if the opening of the acetal ring occurred according to Scheme 2a. The second direction of the acetal ring opening introduces signal “o” and signal “p” in the range 4.47–4.22 ppm. The signals belonging to the ester −CH– groups located at the neighbor of acetal units are observed with a chemical shift of 4.47–4.22 (signal d, k) together with signal “m” from the –CH(CH_3_)–OH end group, as shown in Figure 4.

**Figure 5:**
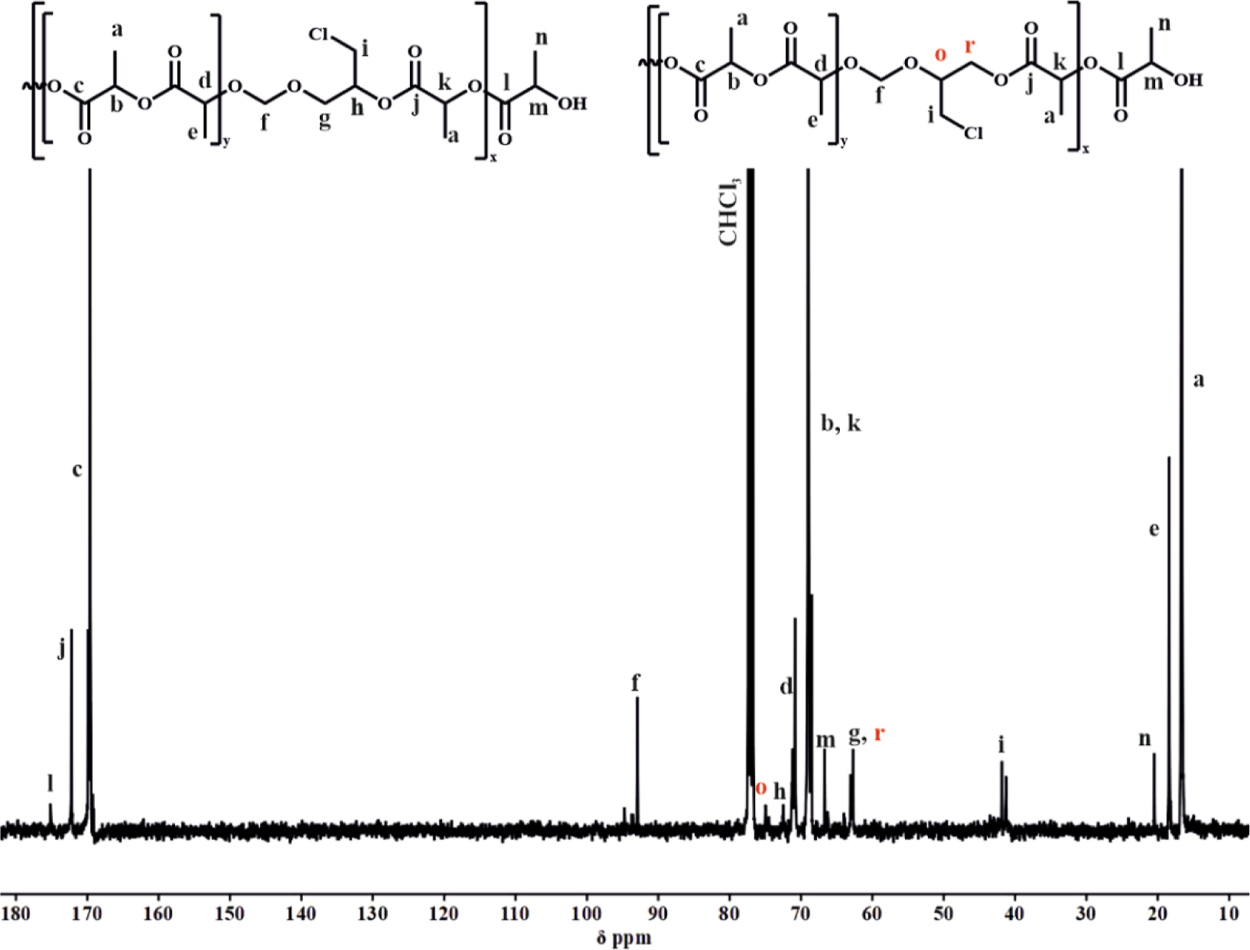
^13^C NMR spectra of PLA/PCl-DXL copolymer with 12 mol % of Cl-DXL (CDCl_3_, 100 MHz).

^13^C NMR (Figure 5) and HSQC NMR (Figure S7) were also used to confirm the presence of both opened acetal units and prove that the protons’ signal was correctly assigned. Two different carbon peaks (−CH_2_Cl, signal (i) at 41.80 and 41.20 ppm) were visible, which correlated with both opened forms of the cyclic acetal in the polymer structure. Additionally, the carbons “g” and “r” or “h” and “o” (−CH– group in the acetal moiety) differ depending on the opening mode of the acetal ring due to the different chemical neighbors, as shown in Figure 5. Moreover, the copolymer structure proved the presence of three different carbon signals at 20.46, 18.40, and 16.62 ppm, corresponding to the –CH_3_ end group, –CH_3_ in heterosequences, and –CH_3_ homosequences, respectively.

**Figure 6:**
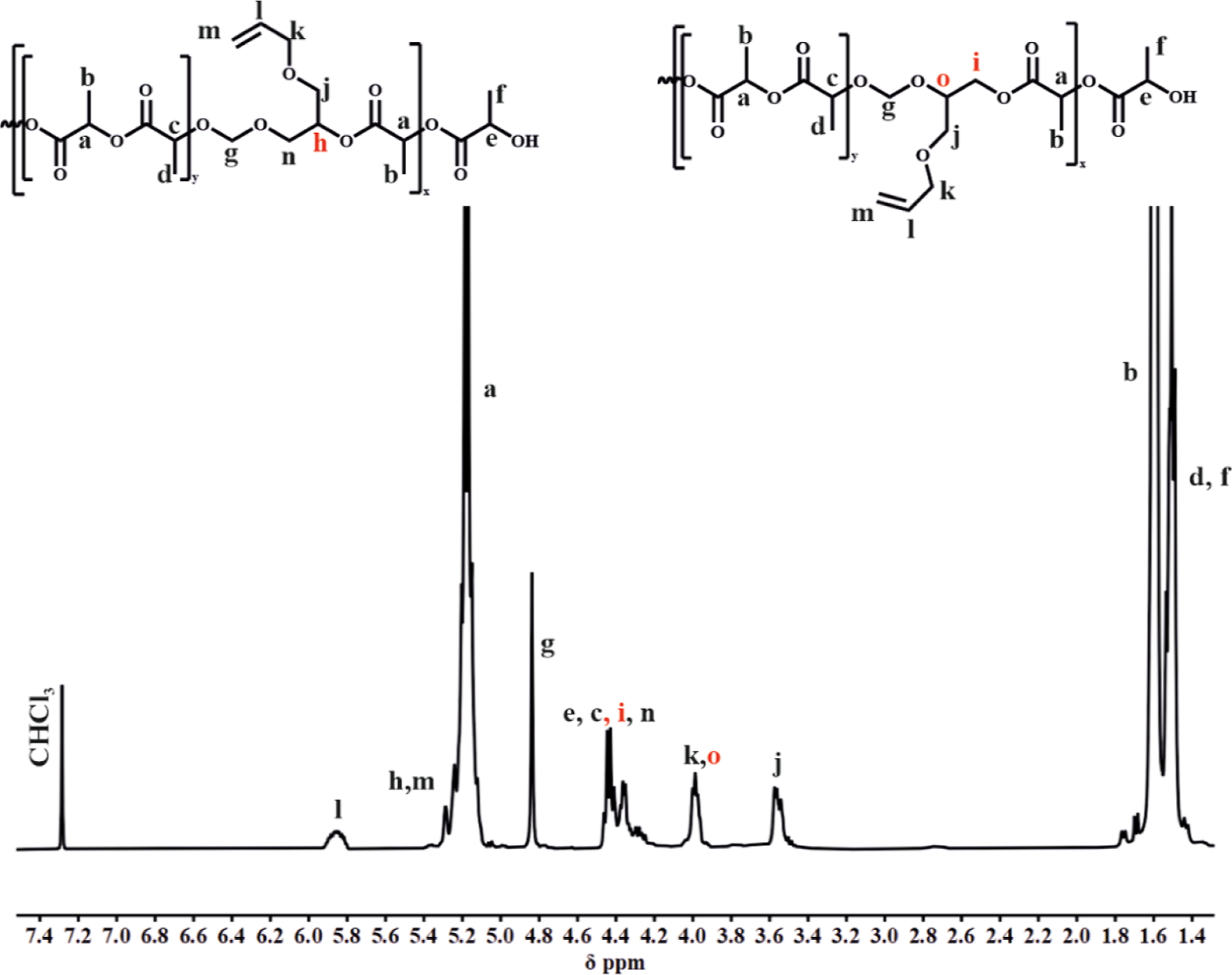
^1^H spectra of PLA/PAllyl-DXL copolymer with 10 mol % Allyl-DXL (CDCl_3_, 400 MHz).

In the ^1^H NMR spectrum of the PLA/PAllyl-DXL copolymers (Figure 6), except for the signals corresponding to the polylactide units, the signals belonging to the oxymethylene and acetal moieties were also visible at 4.81, 3.99, and 3.54 ppm. Signals characteristic of protons located at double bonds were observed at 5.90 and 5.30 ppm. Thus, the ^1^H NMR spectra also revealed that the unsaturated pendant group remained intact under the reaction conditions used, which is vital for further copolymer functionalization.

**Figure 7:**
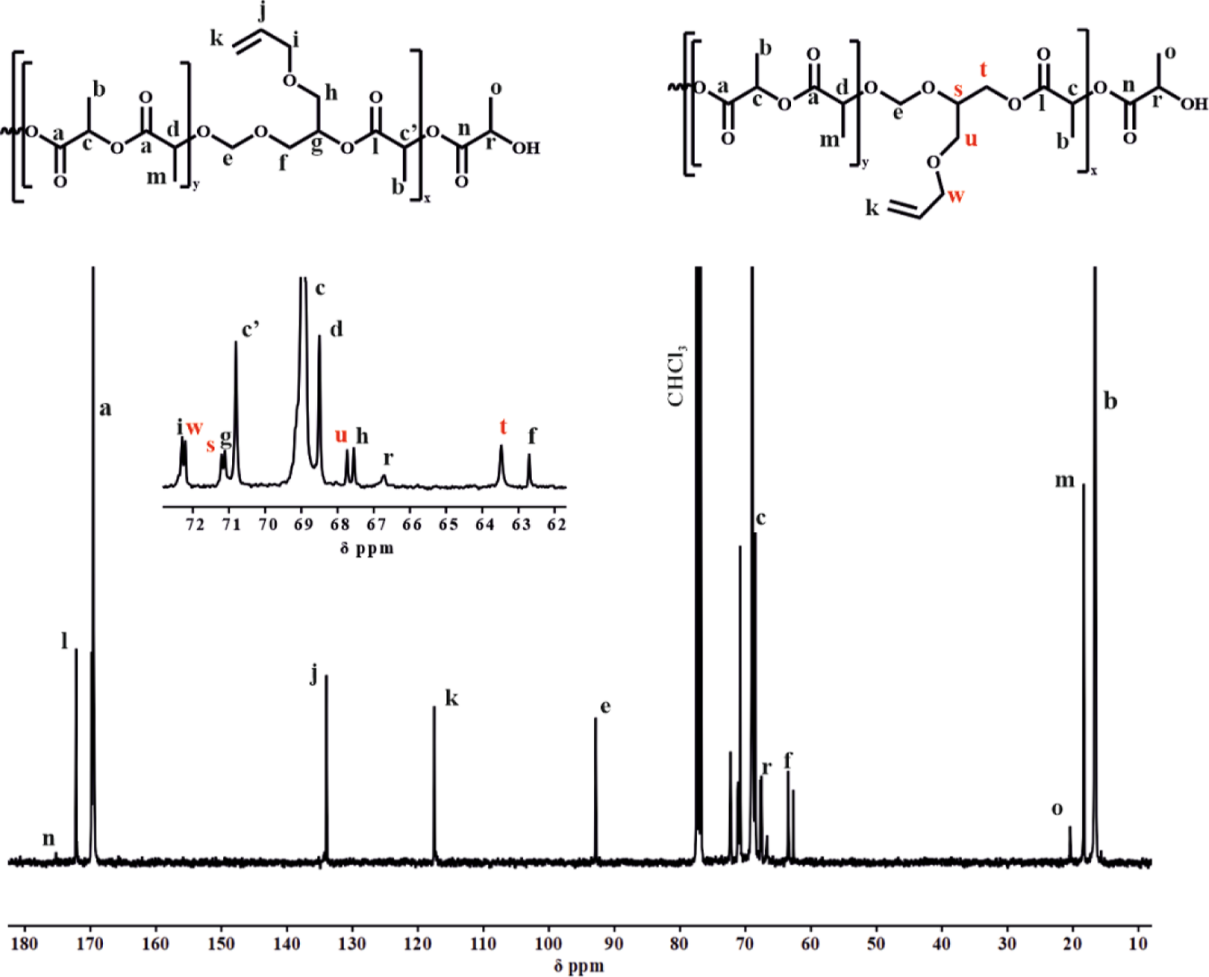
^13^C NMR spectra of the PLA/PAllyl-DXL copolymer with 10 mol % Allyl-DXL (CDCl_3_, 100 MHz).

Similarly as for the PLA/PCl-DXL copolymer, the signals belonging to acetal-ester moieties at 172.18 (carbonyl) and 18.32 ppm (CH_3_– group) appeared in the ^13^C NMR (Figure 7) and 2D HSQC spectra (Figure S8). The –CH_2_– signals corresponding to the acetal moieties occurred in duplicate with a slight difference in chemical shift, which can be attributed to two open ring forms (Scheme 2).

For PLA/PCl-DXL copolymers, the contribution of form “A” or “B” at the polyester chain can be estimated based on the ^1^H NMR spectrum due to the separation of signal “h” from the –CH(CH_2_Cl)– group in the “B” form. The results are summarized in Table S1. The NMR analyses revealed that at 30 °C, the ratio of forms A (52%) and B (48%) in the copolymer chain was comparable. By decreasing the copolymerization temperature from 30 to 2 °C, the content of “A” form decreases from 52 to 43%. In addition, further lowering the temperature to −15 °C did not significantly decrease the content of acetal “A” units (40%) in the copolymer. However, for the PLA/PAllyl-DXL copolymers, these calculations are impossible due to the overlapping of the signals (“m”, “a”, and “o”).

In the case of PLA/PCl-DXL and PLA/PAllyl-DXL copolymers with acetal-ester bonds (due to the inability to homopropagation and formation of acetal–acetal bonds), the sequence length of LA units can be determined taking into account the ratio of the average number of repeating units of the given comonomer (LA /acetal). The obtained values presented in Table 1 indicate that the presence of acetal units significantly disturbed the regularity of the ester sequence and shortened the average length of the PLA block. By changing the polymerization temperature from 30 to −15 °C, the length of the polyester block in the copolyester decreased almost twice.

**Figure 8:**
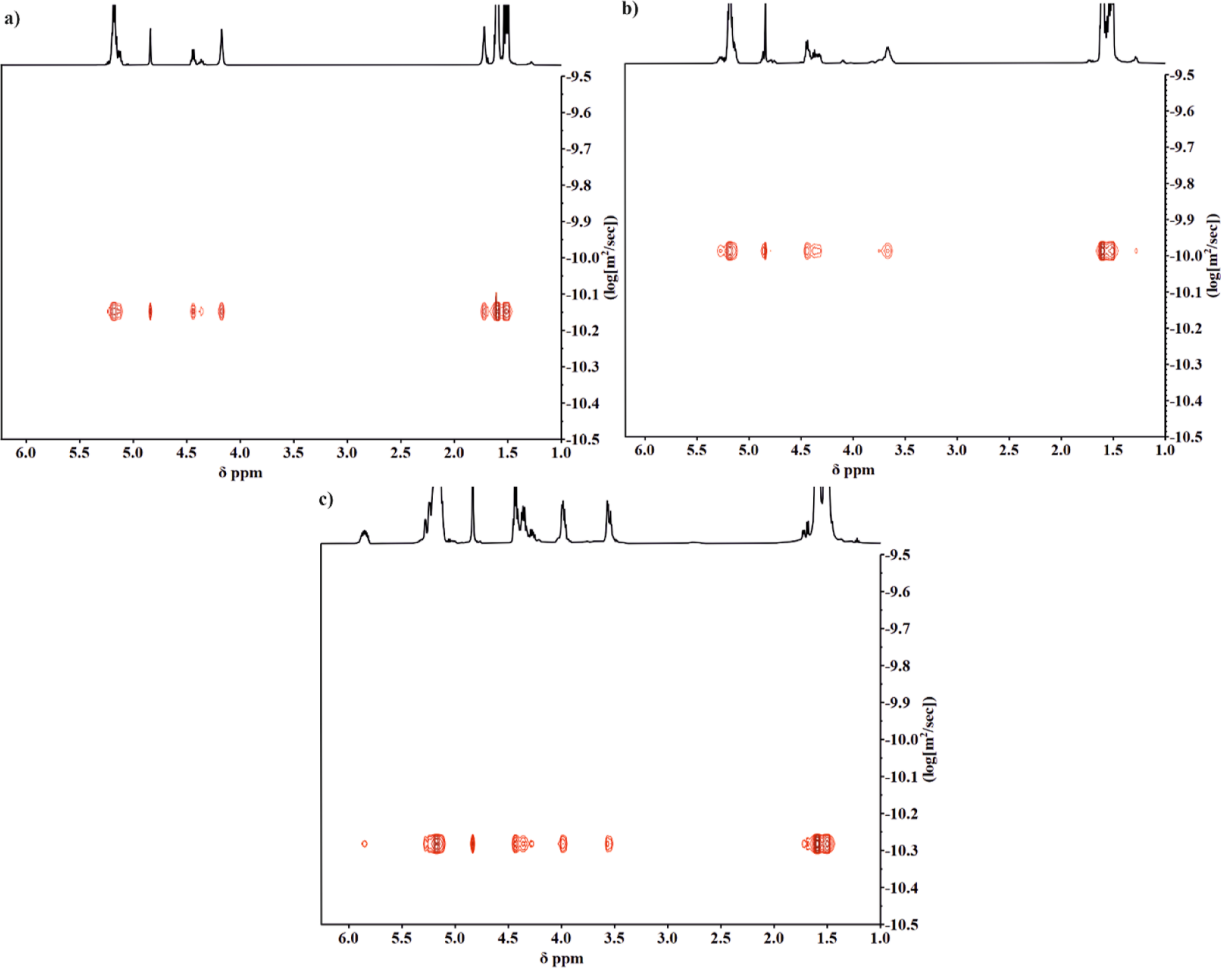
2D DOSY NMR of purified (a) PLA/PDXP_5_, (b) PLA/PCl-DXL, and (c) PLA/PAllyl-DXL copolymers (CDCl_3_, 500 MHz).

For further characterization, diffusion-ordered spectroscopy (DOSY) NMR was employed. This relatively new method enables the structure, composition, molar mass, and diffusion coefficient of analyzed macromolecules to be estimated.^38,39^ The 2D DOSY maps of PLA/PDXP, PLA/PCl-DXL, and PLA/PAllyl-DXL copolymers are shown in Figure 8. The signals belonging to the acetal and ester units in the copolymers exhibited the same diffusion coefficient, which clearly indicates that these moieties are covalently bonded in one macromolecule. The diffusion coefficients were as follows: *D*_PLA/PDXP_ = −10.15, *D*_PLA/PCl-DXL_ = −9.98, and *D*_PLA/PAllyl-DXL_ = −10.28 log(m^2^/s). The other diffusion coefficients of polylactide [*M*_n_ = 10 000 *D* = −9.87 log(m^2^/s)]^40^ or low molar mass compounds were not observed. The DOSY analysis clearly demonstrated that copolyesters containing acetal units were successfully obtained in the cationic copolymerization of lactide with synthesized cyclic acetals.

The presented results confirmed that copolyesters comprising acetal units, which were fitted with reactive functionalities distributed along the copolymer chain, were successfully synthesized. The loading of alkyne and chloride pendant groups may be tuned by the ratio of the comonomers used in the cationic copolymerization. This proves the potential of the presented method as a tool for the effective synthesis of functionalized copolyesters terminated with hydroxyl end-groups under metal-free conditions.

### Modification of the Prepared Polylactides Containing Acetal Functional Units

The pendant reactive groups in the polyester chain provide the opportunity for modification in nucleophilic substitution, azide–alkyne cycloaddition click, or thiol–ene click reactions, as shown in Scheme S2a,b. As a proof of concept, the PLA/PCl-DXL copolymers were reacted with sodium azide to replace the halide group with an azide group.^41,100^ The reaction was performed in DMF or acetonitrile, solvents that are suitable for the S_N_2 mechanism. The modification of copolymers was verified in the ^13^C NMR spectra because of the overlapping proton signal in the ^1^H NMR spectrum. As shown in Figure S9, the signal corresponding to the carbon directly connected to the chlorine ions at 41 ppm disappeared as a new signal from the carbon attached to the –N_3_ at 51 ppm simultaneously appeared. This signal shifted toward a higher frequency due to the deshielding of the carbon by the electronegative azide function.^42^ The SEC analysis (Figure S10a) showed unimodal molar mass distribution and only a slight peak shift toward the lower elution volume due to the small differences between the molar mass of –Cl (35.5 g/mol) and N_3_ (42 g/mol) atoms. We also observed a decrease in the dispersity of azide-functionalized copolymers, which can be correlated with the successive precipitation of the copolymers. The introduction of an azide group to the polymer chain allows for further modification by compounds containing triple bonds.^43^ For this purpose, we used propargyl glycidyl ether or propargyl alcohol to demonstrate that different functional groups may be introduced into copolyesters containing azide functionality through azide–alkyne cycloaddition reactions. The ^13^C NMR showed that after modification, two new characteristic peaks belonging to the triazole ring at 144 and 125 ppm appeared, indicating that the click reaction was successful. Moreover, the spectra in Figure S8 show the presence of new peaks corresponding to the clicked compounds. However, modification of the copolymers with alkyne compounds led to a drastic change in the solubility of the polymers, making GPC analysis in dichloromethane unfeasible.

The presence in the polylactide chain pendant groups with double bonds enables further post modification by thiol–ene “click” chemistry with compounds containing the –SH group.^44^ Compared to internal bonds, external double bonds cause faster and more efficient reactions. A wide range of compounds contain –SH groups, allowing for the introduction of various functionalities, such as carboxylic acid^15^ and hydroxyls. To show the possibility of the copolymer for modification, we used mercaptopropane as a simple nonfunctional thiol to perform a model “click” reaction. As shown in the ^1^H NMR (Figure S11), the conversion of double bonds was complete, as proven by the disappearance of the signal corresponding to the double bond at 5.80 and 5.30 ppm and the appearance of signals at 2.54 and 2.45 ppm, characteristic of –O–CH_2_–CH_2_–CH_2_–S– and −S–CH_2_–CH_2_CH_3_, respectively. Thus, the ^1^H NMR spectra clearly indicated the quantitative yields of the “click” reaction achieved on the copolyester chain. Additionally, to introduce functional groups into the copolymer chain, we reacted mercaptoethanol and thioglycolic acid with a pendant double bond. The representative ^1^H NMR spectra of copolymers before and after the thiol–ene reaction are shown in Figure S11. For all of the reactions performed, the ^1^H NMR results confirmed the addition of thiol molecules to the alkyne groups distributed along the polyester chain. These reactions resulted in the preparation of copolyesters with hydroxyl and carboxyl pendant groups. GPC analysis of “clicked” copolymers is in accordance with ^1^H NMR, as the trace of the product almost exhibited the same shape as the trace of PLA/PAllyl-DXL before the modification but slightly shifted to lower elution volume values (higher molar mass), as shown in Figure S10b. The GPC analysis of the copolymers modified by thioglycolic acid was not possible due to the strong interaction of the carboxylic group with the PLGel chromatography column.

Most importantly, the applied modification conditions did not cause the copolymer chain containing acetal–ester bonds to degrade, which was proven by ^1^H NMR analysis. Thus, this wide variety of functionalization reactions demonstrated that the synthesized copolyesters could be easily modified under mild conditions with a high yield by various functional and nonfunctional compounds.

### Thermal Properties of Prepared Copolymers

The PLA/PDXP, PLA/PCl-DXL, and PLA/PAllyl-DXL represent a novel category of polymers distinguished from unmodified PLA due to the incorporation of diverse acetal units. In order to gain insights into the influence of these acetal units on the thermal properties and stability of the copolyesters, we conducted TGA (thermogravimetric analysis) and DSC (differential scanning calorimetry) analyses. Initially, we investigated the crystallization behavior of both the initial copolymers and their modified counterparts during the second DSC heating cycle. The DSC and TGA outcomes of the unmodified copolymers are summarized in Table 2 and Figures S12a–c and S13a–c.

**Table 2:** Thermal Properties of the Starting, Purified Copolymers Determined from TGA and DSC

		DSC		TGA
run	sample	*T*_g_ (°C)	*T*_m_ (°C)	*T*_max_ (°C)
	PLA^22^	60	160	300
1	PDXP	–78	16	350
2	PLA/PDXP_5_	30		351
3	PLA/PDXP_10_	22		318
4	PLA/PDXP_18_	17		330
5	PLA/PDXP_26_	6		357
10	PLA/PCl-DXL_10_	40		320
11	PLA/PCl-DXL_16_	35		350
12	PLA/PCl-DXL_22_	25		355
17	PLA/PAllyl-DXL_10_	38		340
18	PLA/PAllyl-DXL_14_	27		310
19	PLA/PAllyl-DXL_22_	14		327

Neat PDXP exhibited a glass transition (*T*_g_) below −78 °C and the melting temperature (*T*_m_) at 16 °C. The semicrystalline PLA exhibits a *T*_g_ of approximately 60 °C and a *T*_m_ up to 180 °C, depending on the molar masses.^45^ As shown in Table 2, *T*_g_ and the crystallization capability were affected by the introduction of acetal units in all of the analyzed copolymers, regardless of their type. In all instances, the observation of the melting temperature peak was absent, suggesting that the copolyesters prepared are highly amorphous. This observation is in agreement with our previous studies,^22^ that revealed that the incorporation of dioxolane units into the polylactide chain completely eliminated the crystallization of copolymer.

For copolymers of lactide with dioxepane and functionalized acetals, it is also noticeable that the increase in the number of acetal units in the polyester chain leads to a gradual decrease in *T*_g_. The obtained copolymers exhibit *T*_g_ values ranging from 40 to 6 °C, depending on their structure and acetal content. The PLA/PDXP copolymer with 26% acetal content displays the lowest *T*_g_, which could potentially be attributed to the presence of homopolydioxepane sequences.^22^

The thermal stability of the resulting copolymers was studied using thermogravimetric analysis with a heating rate of 20 °C/min from 30 to 650 °C. The results in Table 2 show that the temperature of the maximum rate of decomposition value increased by 50° in comparison to PLA, and it is close to the *T*_max_ of neat PDXP. The TGA results clearly indicate that the introduction of acetal units contributes to the modification of the thermal stability of polylactide.

The results of the modified copolyesters thermal analysis are summarized in Table 3. A comparison of the DSC scans for the starting and the corresponding modified copolymers showed that the incorporation of various functional groups to the copolymer chain did not affect the thermal properties and stability significantly. The modification only resulted in a slight decrease in the *T*_g_ value compared to the nonmodified copolymers, from 38 to 30 °C. The TGA results showed that only the copolymer modified with thioglycolic acid had a lower *T*_max_ compared to the nonmodified one. This effect can be correlated with the presence of the pendant carboxylic acid groups that can undergo condensation or decarboxylation upon thermal heating.^46^

**Table 3:** Characteristic Temperatures Determined from TGA and DSC of the Modified Copolymers in Comparison to the Starting Copolyester

		DSC		TGA
sample	modifying agent	*T*_g_ (°C)	*T*_m_ (°C)	*T*_max_ (°C)
PLA/PAllyl-DXL_10_		38		340
PLA/PAllyl-DXL_10_	propanethiol	35		324
PLA/PAllyl-DXL_10_	thioglycolic acid	31		302
PLA/PAllyl-DXL_10_	mercaptoethanol	30		324
PLA/PCl-DXL_10_		40		320
PLA/PCl-DXL_10_	sodium azide	34		350
PLA/PCl-DXL_10_	sodium azide/propargyl glycidyl ether	32		300

### Acid-Triggered Degradation of Functionalized Polylactides Containing Acetal Units

In our previously published work,^22^ we proved that the random distribution of acetal units (27 mol %) in the polylactide chain enabled the degradation of copolymers within 72 h.

**Figure 9:**
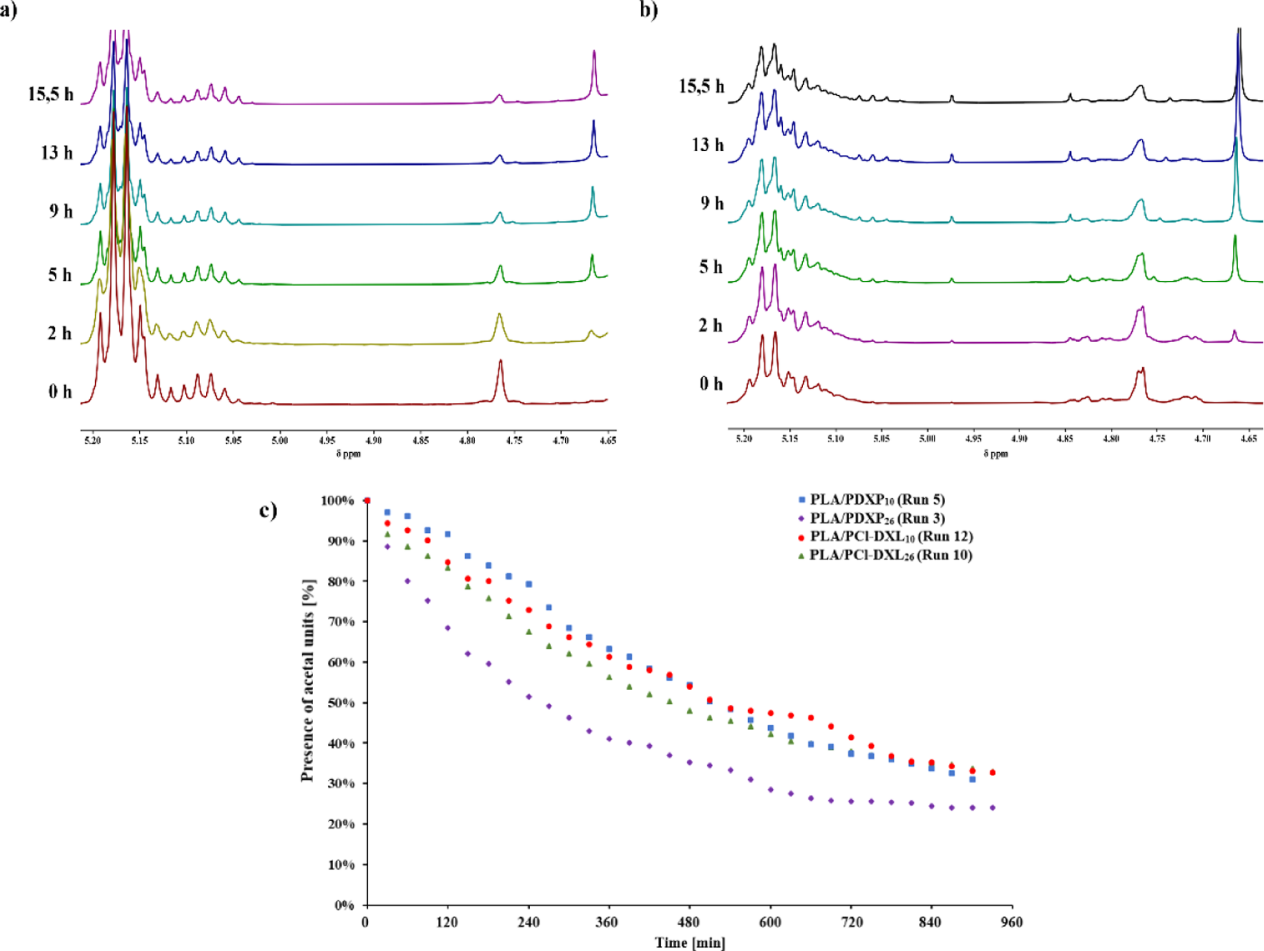
Acid-triggered degradation of (a) PLA/PDXP_10_, (b) PLA/PCl-DXL_10_ copolymers, and (c) disappearance of the signal attributed to –OCH_2_O– units in the copolymer chains (500 MHz, CD_3_CN, solution of HCl in CD_3_CN, see experimental part).

In the present paper, to show the possibility of the copolymer degradation in acidic medium, we selected for experiments nonfunctionalized (PLA/PDXP) and chloro-functionalized (PLA/PCl-DXL) copolyesters. The progress of the copolymer chain cleavage was studied using ^1^H NMR spectra (Figure 9). As we showed for the lactide copolymers with 1,3-dioxolane,^22^ the presence of the acetal-ester bond requires harsher conditions to initiate the hydrolysis than a typical acetal bond.^23^ For this purpose, we applied a hydrolytic system composed of a water solution of HCl (35%) and deuterated acetonitrile at 40 °C. This solvent combination allows to study degradation of copolymers in a homogeneous system, whereby the cleavage of acetal-ester bonds can be traced in the ^1^H NMR spectra due to the good separation of signals. For the analyzed copolymers, the initial ^1^H NMR spectra were recorded immediately after acid was added to the NMR tube and after a selected time interval.

First, we monitored changes in the spectra of lactide copolymers containing 10 mol % of acetal units. As shown in Figure 9a (PLA/PDXP_10_) and Figure 9b (PLA/PCl-DXL_10_) the intensity of the signal attributed to the –OCH_2_O– protons in an acetal–ester bond (4.78 ppm) gradually decreased over time, indicating the bond cleavage in the copolymer chain. At the same time, the intensity of signals in the region of spectra 5.03–5.20 did not change significantly, which allows us to conclude that there was no degradation of ester bonds in the LA–LA homosequences. Such an observation was also reported earlier for copolymers of lactide with 1,3-dioxolane under the conditions used [10 μL HCl (35%) in 690 μL CD_3_CN].^22^ At the same time, a new signal in the spectra appeared at 4.67 ppm, and its intensity grew with time. It can therefore be assumed that the presence of this signal may be related to the formation of population polymer chains with new end-groups or byproducts in the system. Identifying all the degradation products in the studied system is cumbersome due to the presence of substitution on the cyclic acetal (two ways of opening the acetal ring) and nonclassical acetal bonds (acetal–ester bond) in the copolymer chain. However, our group is currently conducting a more complex investigation to elucidate the mechanism of acid-triggered degradation of copolyesters containing various types of acetal units.

For copolymers differing in composition, we determined the rate of disappearance of signal located at 4.78 ppm, by analyzing its intensity (*I*) in time, expressed as the *I*_*t*_/*I*_0_ ratio (Figure 9c). The provided graph clearly demonstrates that, under the specified conditions, the 50% reduction in signal intensity was achieved after 270 min (4.5 h) for PLA/PDXP_26_ (run 5), and about 510 min (8 h) for PLA/PDXP_10_ (run 3), PLA/PCl-DXL_22_ (run 12), and PLA/PCl-DXL_10_ (run 10). Thus, the fastest degradation was observed for the copolymer containing the highest content of DXP–DXP sequences in the copolymer chain (run 5), which are more sensitive to pH than the acetal–ester bonds present in the other analyzed copolymers.

No significant difference in the rate of acetal unit loss was observed between chloro-functionalized PLA containing 10 and 20 mol % of acetal (runs 10 and 12). The increased content of Cl-DXL units within the polyester chains did not result in faster degradation since only acetal-ester bonds were present in this copolymer type. The slight differences in the degradation times between PLA/PDXL and PLA/PCl-DXL could be associated with the different hydrophobicities of the prepared materials. This effect was investigated by comparing the contact angle measured for PLA and selected copolymers, as shown in Figure S14. The introduction of various acetal units to the polyester chain caused a reduction in the contact angle from 85° (PLA) to 76° (PLA/PDXP) and 62° (PLA/PCl-DXL). These results are consistent with the degradation experiments, which indicated that PLA/PCl-DXL degrades slightly faster than PLA/PDXP, likely due to its higher hydrophilicity.

Additionally, to demonstrate the loss of copolymers’ molar mass after acid-triggered degradation under the applied conditions, we performed SEC analysis. As shown in Figure S15 for PLA/PDXP_10_ and PLA/PCl-DXL_10_, the SEC traces showed a decrease in molar masses from 10 000 to 3 000 and from 13 000 to 3 500 g/mol, respectively, within 24 h. The decrease in molar masses of the copolymers was attributed to the cleavage of the labile acetal–ester bonds in the main chain.

## Conclusions

In this work, we have successfully synthesized functionalized polylactides bearing chloride and allyl functions as side groups via cationic ring-opening copolymerization of lactide with the appropriate cyclic acetals. The strategy allowed a high content of functional pendant groups to be incorporated along the copolymer backbone during the polymerization process. The copolymer composition was controlled by changes in the copolymerization parameters or the feed ratio, and the final amount of cyclic acetals in the polylactide chain ranged from 5 to 27 mol %. Subsequently, nucleophilic substitution, thiol–ene, and azide–alkyne click reactions have been applied to modify the copolyester chain by reacting the pendant groups with sodium azide, propargyl alcohol, mercaptopropane, mercaptoethanol, or thioglycolic acid. The results obtained from the hydrolysis studies showed that the introduction of acetal units into the macromolecules led to the cleavage of the copolymer chain under relatively mild acid conditions. In summary, the described procedure allowed for the synthesis of functionalized polylactides with a controlled number of side functional groups, thanks to which it is possible further to construct more complex, polyester-based degradable structures.

## Supporting Information Available

^1^H, ^13^C NMR, and GC/MS spectra of comonomers; ^1^H, ^13^C, 2D HSQC NMR spectra of copolymers; SEC traces of starting and modified copolymers; contact angles of copolymers; scheme of synthesis of comonomers; and modification of the copolymers (PDF)
