# Supplementary material for: Synthesis and Characterization of Functionalized Polylactides Containing Acetal Units
Source: Macromolecules. 2023 Aug 16;56(17):6951–67. doi: 10.1021/acs.macromol.3c01343 (PMC10501204; doi:10.1021/acs.macromol.3c01343)
Supplement: Supplementary file 1 — ma3c01343_si_001.pdf [file ma3c01343_si_001.pdf]

Supporting information for

Synthesis and characterization of functionalized polylactides containing acetal units

Karolina Cichoń, Irena I. Bak-Sypien, Malgorzata Basko*, Bartłomiej Kost*

Centre of Molecular and Macromolecular Studies Polish Academy of Sciences

Sienkiewicza 112, 90-363 Lodz, Poland

Emails: bartlomiej.kost@cbmm.lodz.pl, malgorzata.basko@cbmm.lodz.pl

Table of contents:

Table S1. The molar content of two different types of acetal repeating units in PLA/PCl-DXL_{12} copolymer chain calculated from the ^{1}H NMR spectra, taking into account the intensity of signal "h" and "f"…………………………………………………………………..………... 3

Scheme S1. The synthesis of cyclic acetals via a dehydration reaction ……………………… 3

Figure S1. The ^{1}H NMR spectra of a) 1,3-dioxepane, b) 4-chloromethyl-1,-3-dioxolane, c) 4-[(allyloxy)methyl]-1,3-dioxolane …………………………………………………… 4

Figure S2. The ^{13}C NMR spectra of a) 1,3-dioxepane, b) 4-chloromethyl-1,-3-dioxolane, c) 4-[(allyloxy)methyl]-1,3-dioxolane ……………………………………………...…….. 4

Figure S3. a) the GC chromatogram of obtained cyclic acetals and b) 4- chloromethyl-1,3-dioxolane c) 1,4-dioxepane, and d) 4-[(allyloxy)methyl]-1,3-dioxolane MS spectra ………… 5

Figure S4. a) ^{1}H NMR spectra and b) ^{13}C NMR spectra of poly(1,3-dioxepane) homopolymer ………………………………………………………………………………… 6

Figure S5. a) 2D NMR HSQC spectra of PLA/$PDXP_5$ copolymer ($CDCl_3$, 400 MHz) ……. 6

Figure S6. Conversion vs time curves for cationic copolymerization of lactide with Cl-DXL…………………………………..…...……………………………………………….... 7

Figure S7. a) 2D NMR HSQC spectra of PLA/PCl-DXL copolymer ($CDCl_3$, 400 MHz) ….. 8

Figure S8. a) 2D NMR HSQC spectra of PLA/PAllyl-DXL copolymer ($CDCl_3$, 400 MHz)... 8

Scheme S2. Functionalization of a) PLA/PCl-DXL copolymer with sodium azide, propargyl alcohol or propargyl glycidyl ether and b) PLA/PAllyl-DXL copolymer with propanethiol, glycolic acid or ethanethiol………………...……………………………………………….. 9

Figure S9. Figure S8. The ^{13}C NMR spectra before and after modification of PLA/PCl-DXL (DMSO-d6, 100 MHz) ……………………………………………………….………… 10

Figure S10. The SEC trace of PLA/PCl-DXL and PLA/PAllyl-DXL before and after modification…………………………………………………………………………….. 11

Figure S11. The ^{1}H NMR spectra before and after modification of PLA/PAllyl-DXL (CDCl3, 400 MHz) ………………………..…………………………………………..………... 12

Figure S12. The DSC traces of a) PLA/PDXP, b) PLA/PCl-DXL or PLA/Pallyl-DXL copolymers, and c) DSC traces of copolymers after modification…………………………… 12

Figure S13. The TGA traces of a) PLA/PDXP, b) PLA/PCl-DXL or PLA/Pallyl-DXL copolymers, and c) DSC traces of copolymers after modification…………………………… 13

Figure S14. Contact angles measured for neat PLA and PLA/PDXL and PLA/PCl-DXL copolymers (films on a glass plate with water as a reference liquid)……………………….. 13

Figure S15. The GPC traces of a) PLA/PDXP and b) PLA/PCl-DXL copolymers before and after hydrolysis…………………………………………………………………….... 14

Table S1. The molar content of two different types of acetal repeating units in PLA/PCl-DXL$_{12}$ copolymer chain calculated from the ^{1}H-NMR spectra taking into account the intensity of signal "h" and "f"

Sample	Temperature	„A" form [mol%]	„B" form [mol%]
PLA/PCl-DXL$_{12}$	30	52	48
PLA/PCl-DXL$_{18}$	2	43	57
PLA/PCl-DXL$_{22}$	-15	40	60

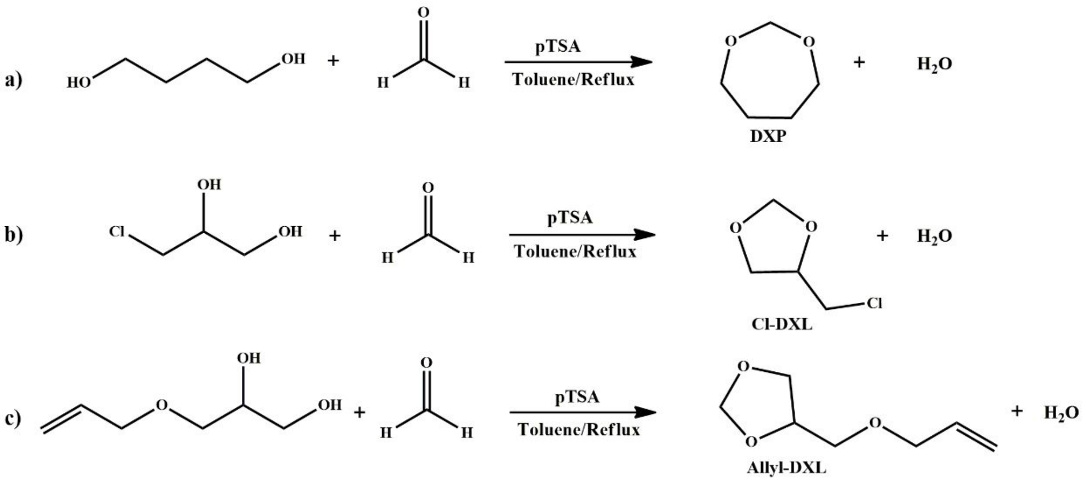

Scheme S1. The synthesis of cyclic acetals a) 1,3-dioxpeane (DXP), b) 4-chloromethyl-1,3-dioxolane, c) 4-[(allyloxy)methyl]-1,3-dioxolane

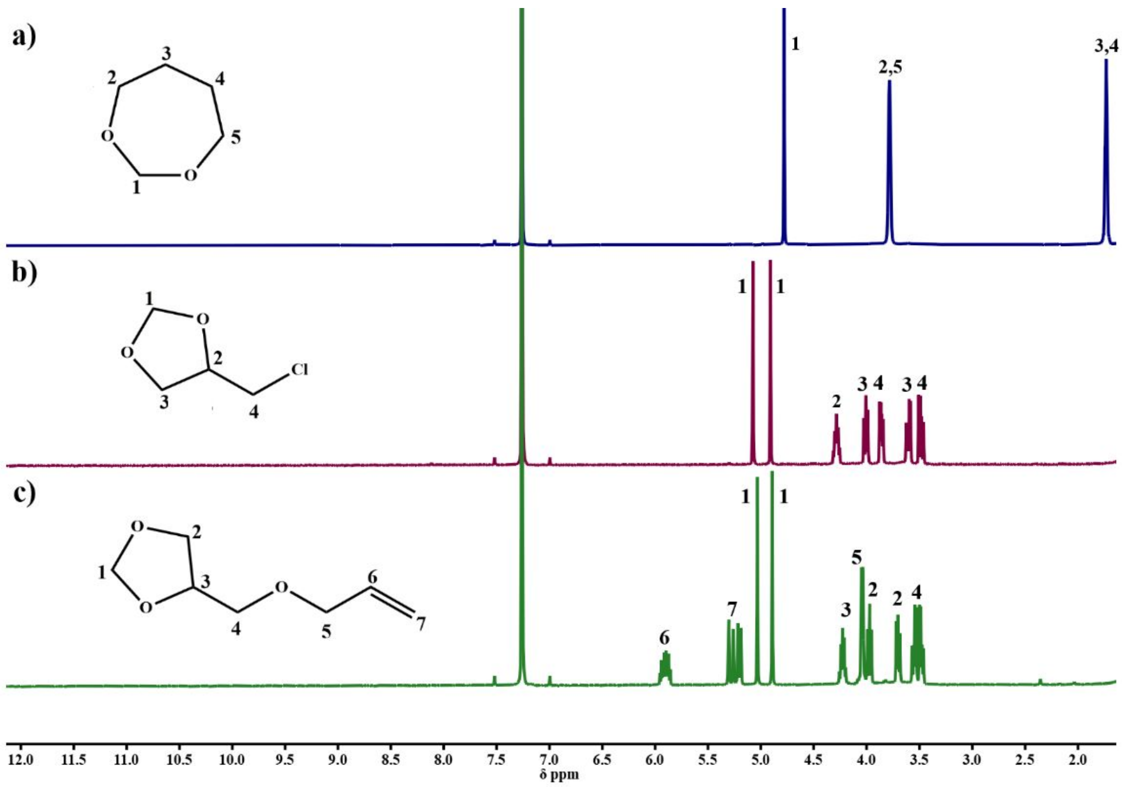

Figure S1. The ^{1}H NMR spectra of a) 1,3-dioxepane, b) 4-chloromethyl-1,3-dioxolane, c) 4-[(allyloxy)methyl]-1,3-dioxolane

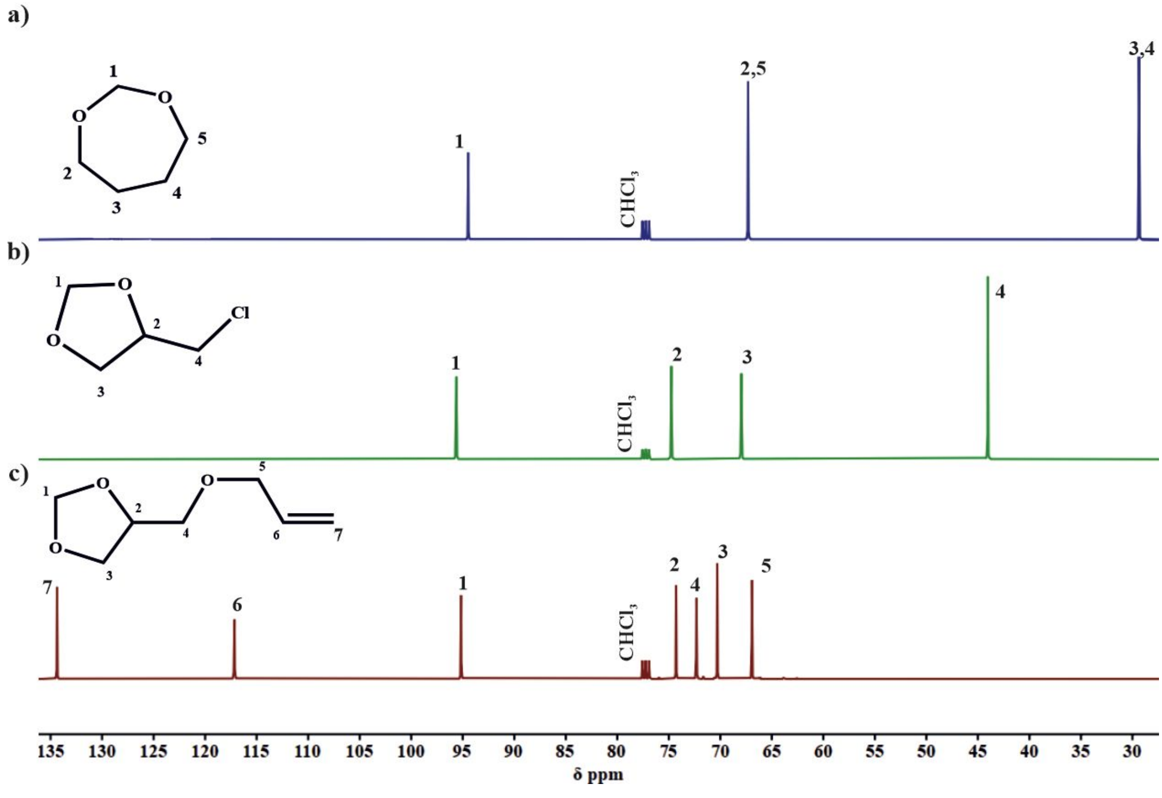

Figure S2. The ^{13}C NMR spectra of a) 1,3-dioxepane, b) 4-chloromethyl-1,3-dioxolane, c) 4-[(allyloxy)methyl]-1,3-dioxolane

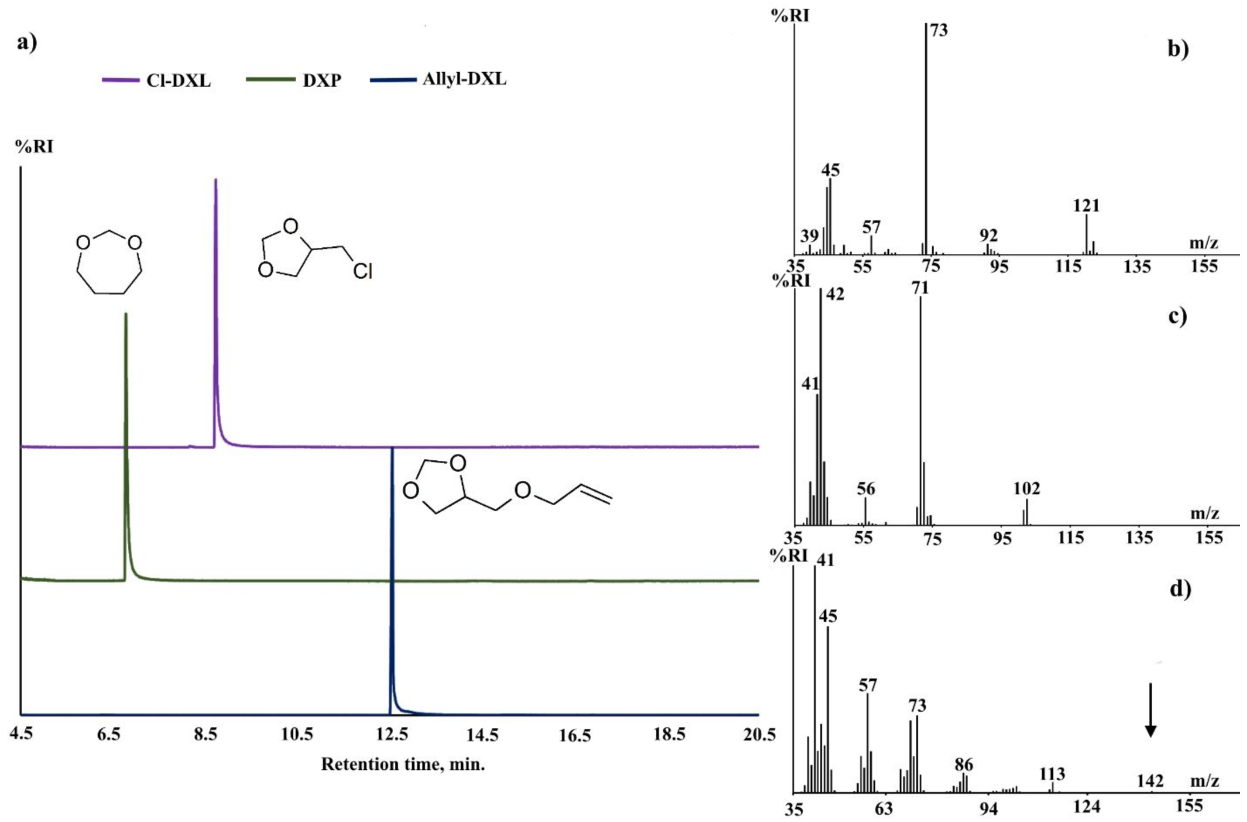

Figure S3. a) the GC chromatogram of obtained cyclic acetals and b) 4 -chloromethyl-1,3-dioxolane (Cl-DXL), c) 1,3-dioxepane (DXP), and d) 4-[(allyloxy)methyl]-1,3-dioxolane (Allyl-DXL) MS spectra

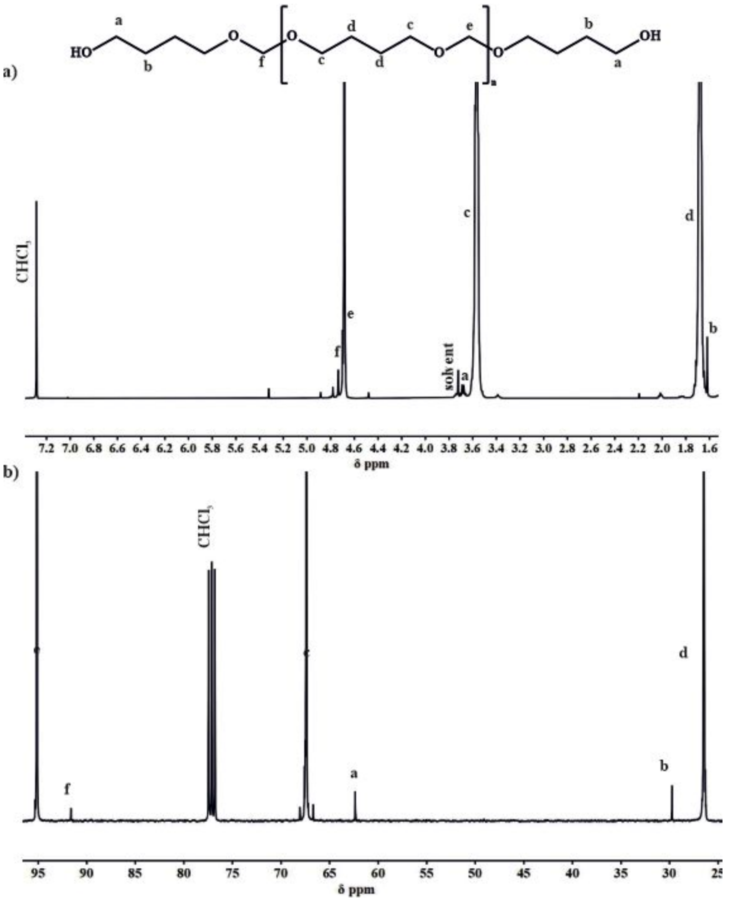

Figure S4. a) ^{1}H NMR spectra and b) ^{13}C NMR spectra of poly(1,3-dioxepane) homopolymer

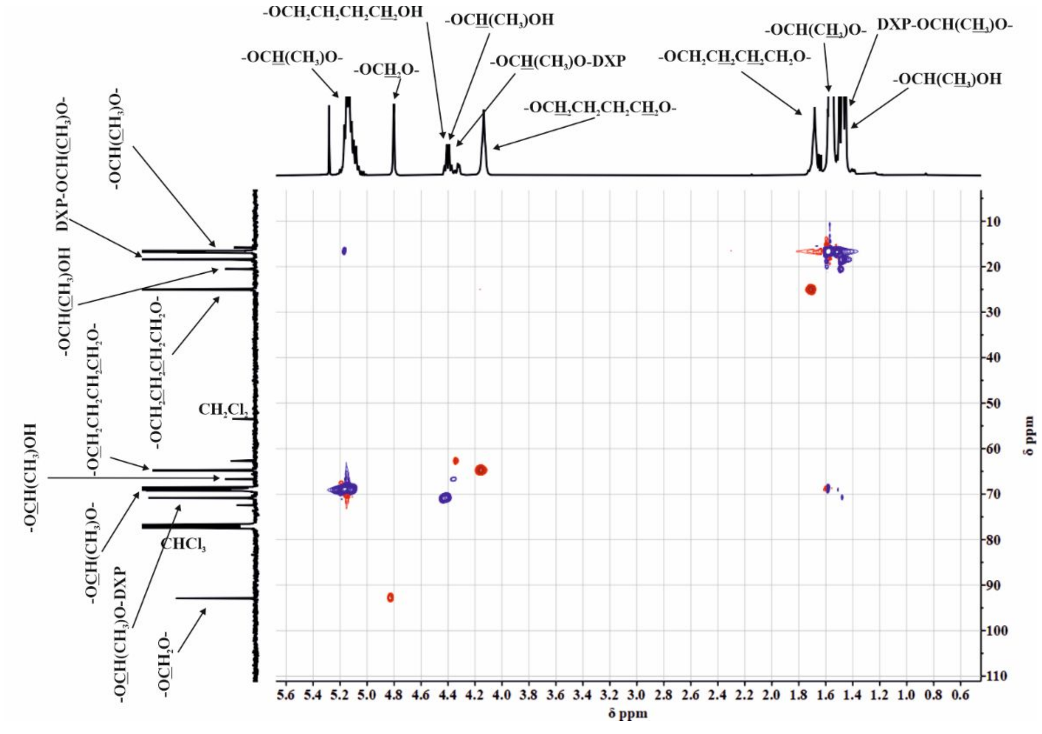

Figure S5. a) 2D NMR HSQC spectra of PLA/PDXP$_5$ copolymer ($CDCl_3$, 400 MHz)

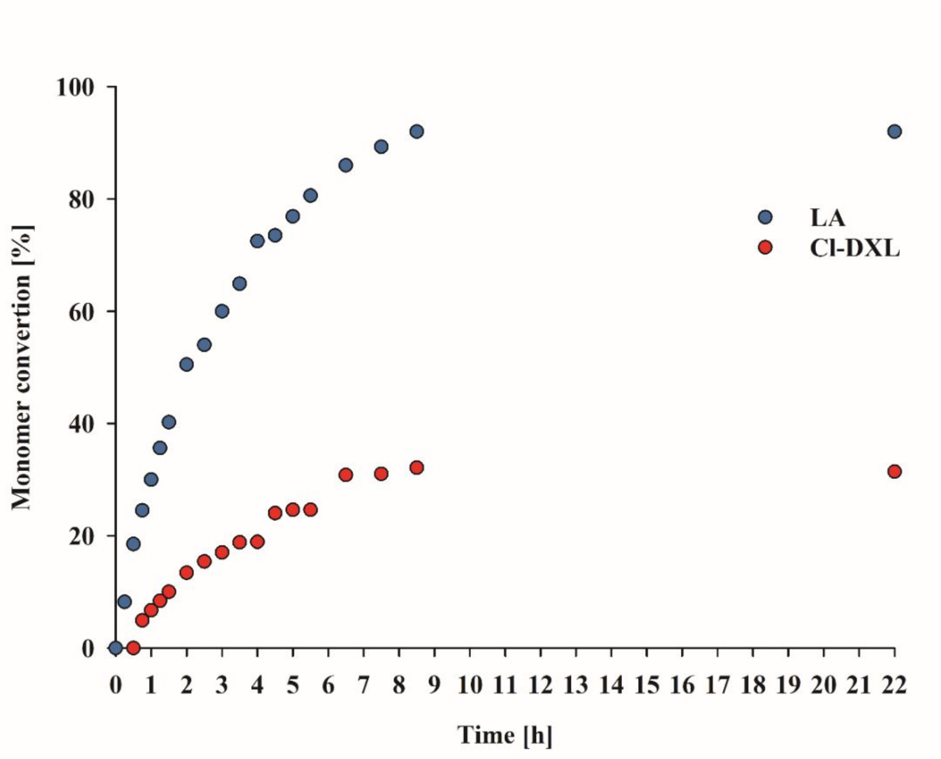

Figure S6. Conversion vs time curves for cationic copolymerization of lactide with Cl-DXL. Conditions: (LA/ Cl-DXL =67/33 mol%, ethylene glycol as an initiator, triflic acid as a catalyst, T= 2 ^{o}C, run 11 at Table 1).

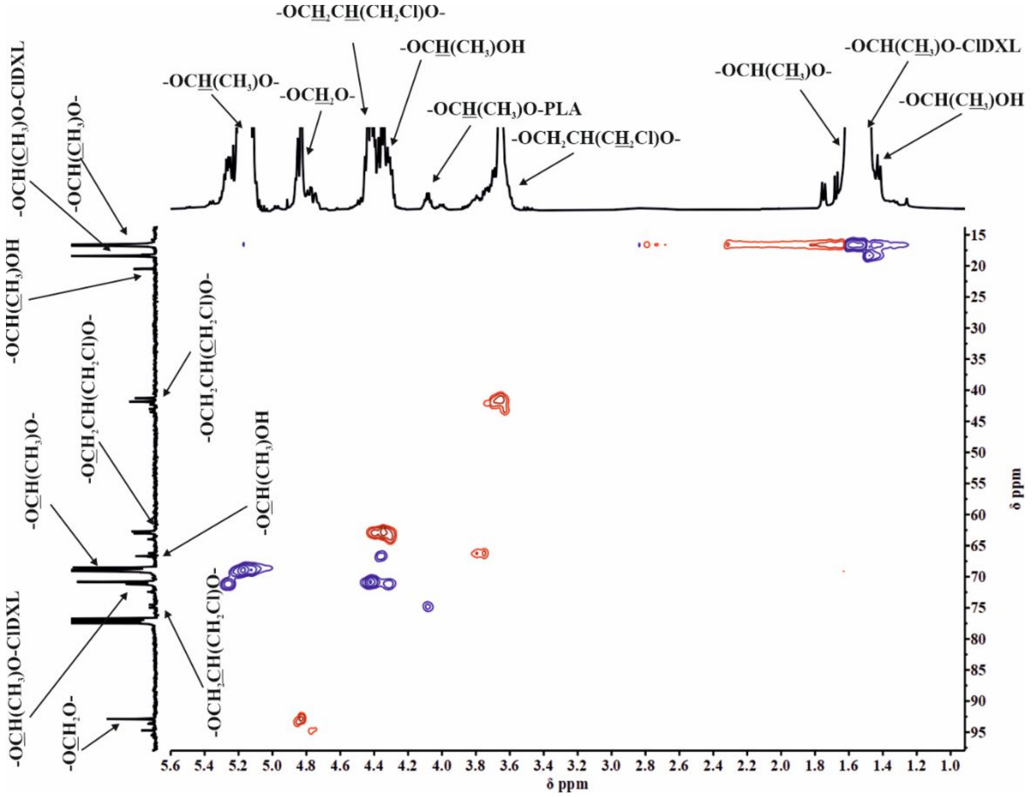

Figure S7. a) 2D NMR HSQC spectra of PLA/PCl-DXL copolymer ($CDCl_3$, 400 MHz)

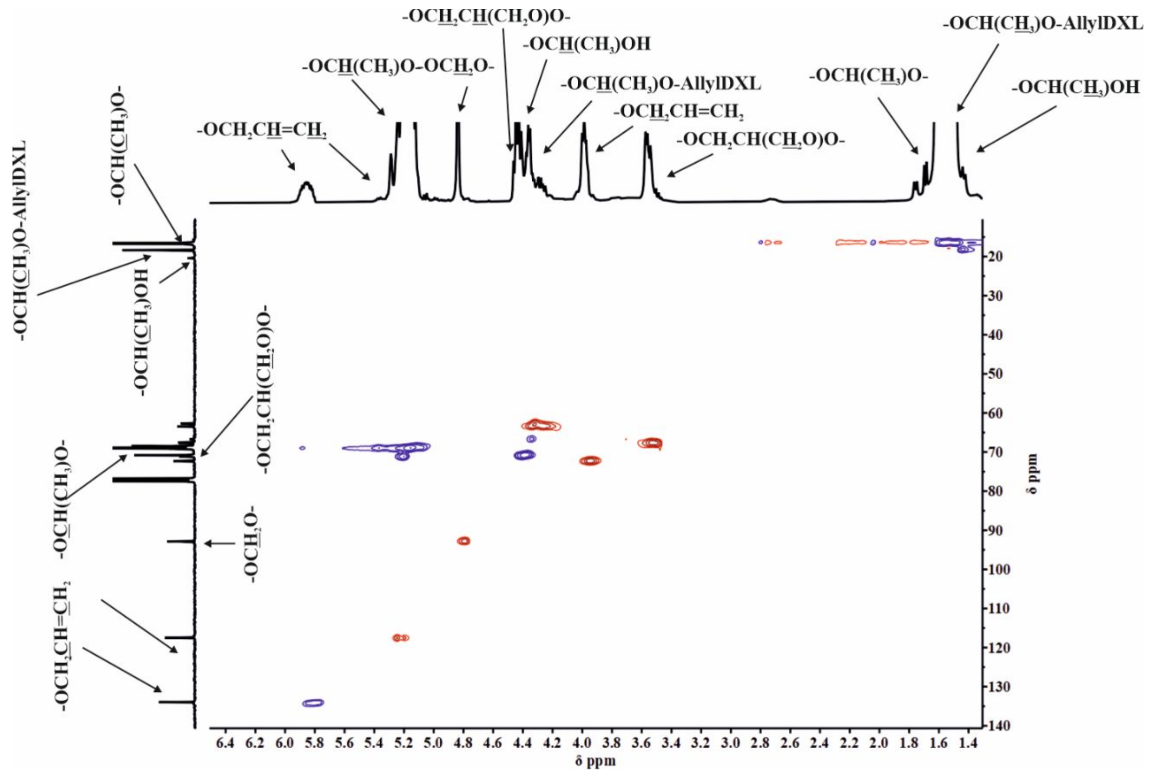

Figure S8. a) 2D NMR HSQC spectra of PLA/PAllyl-DXL copolymer ($CDCl_3$, 400 MHz)

a)

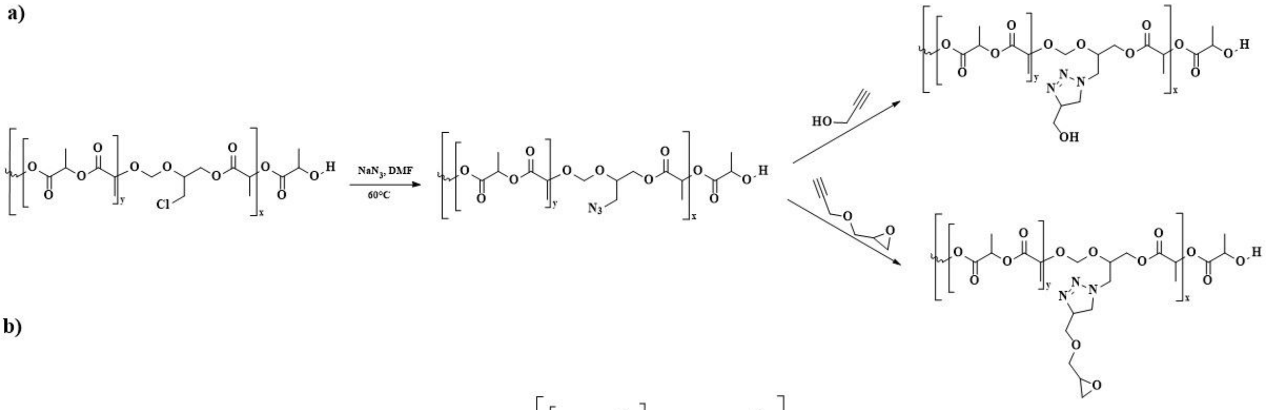

b)

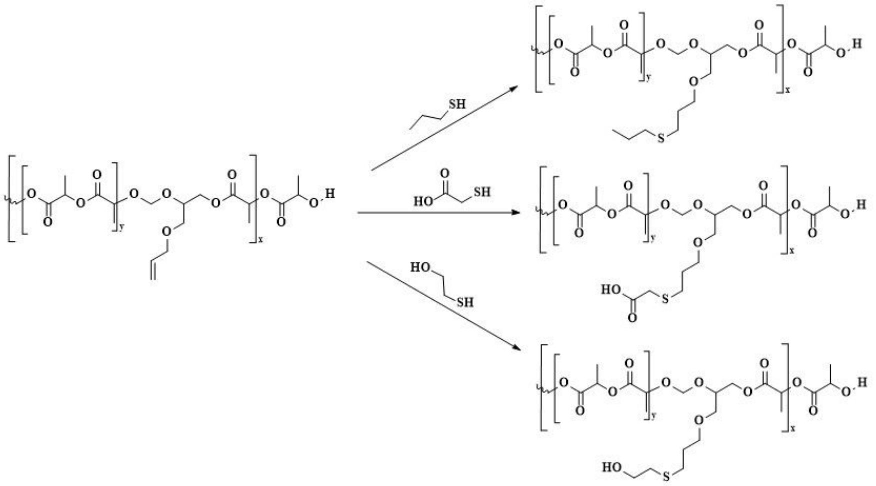

Scheme S2. Functionalization of a) PLA/PCl-DXL with sodium azide, propargyl alcohol or propargyl glycidyl ether and b) PLA/PAllyl-DXL with propanethiol, glycolic acid or ethanethiol

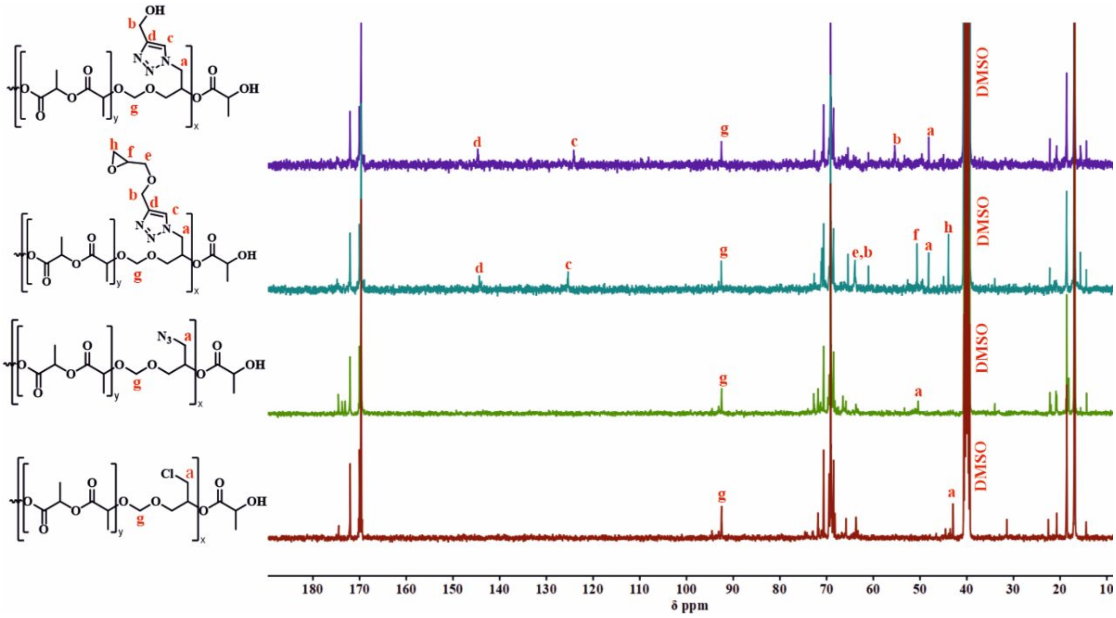

Figure S9. The ^{13}C NMR spectra before and after modification of PLA/PCl-DXL (DMSO-d_6, 100 MHz)

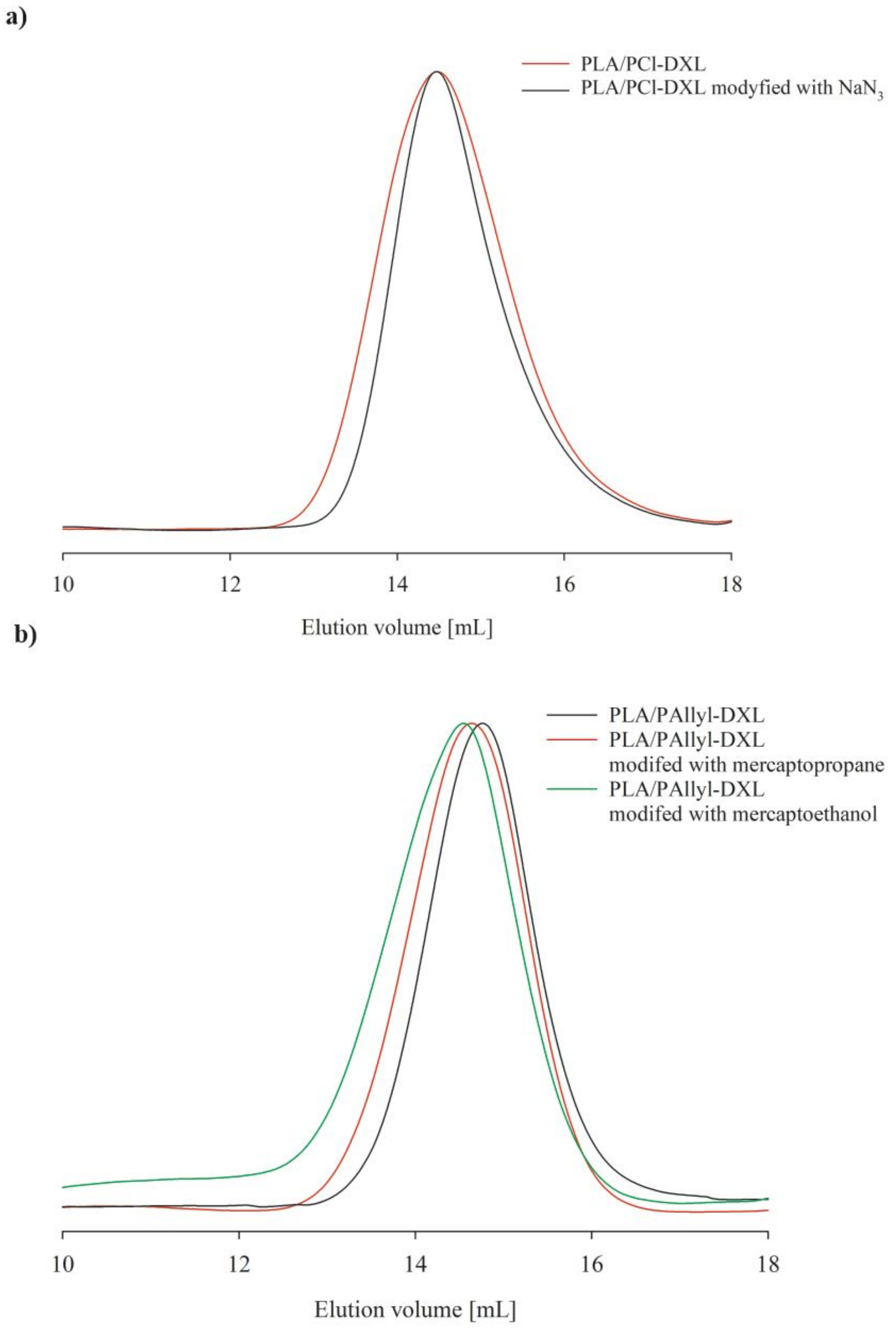

Figure S10. The SEC trace of PLA/PCl-DXL and PLA/PAllyl-DXL before and after modification

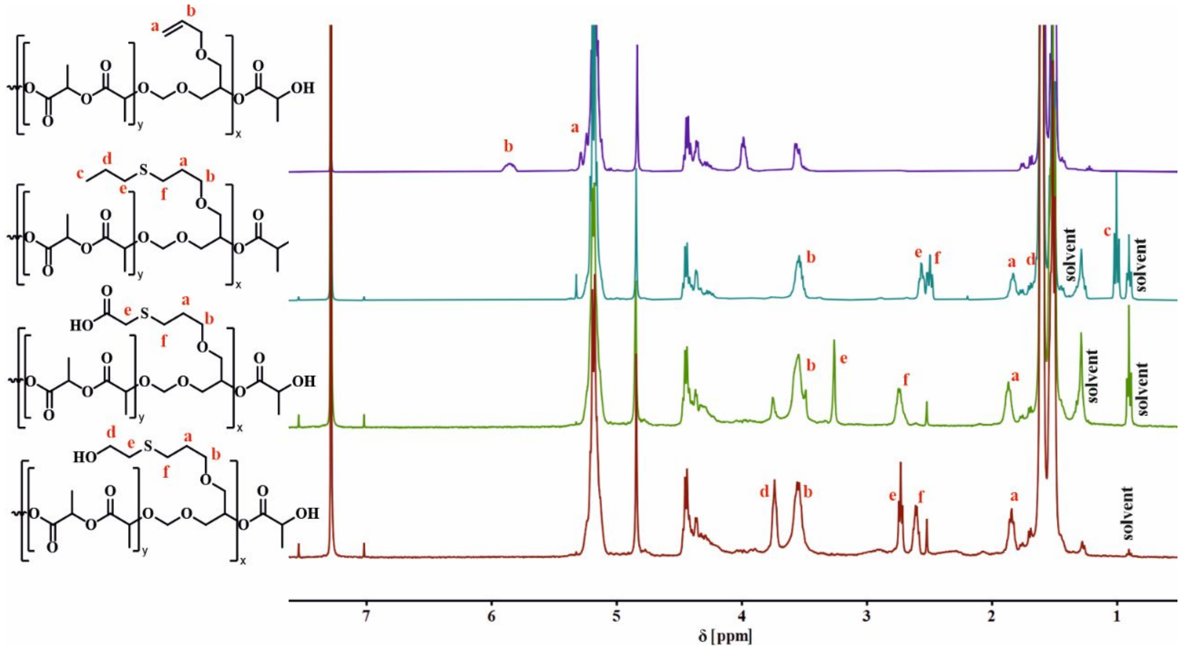

Figure S11. The ^{1}H NMR spectra before and after modification of PLA/PAllyl-DXL ($CDCl_3$, 400 MHz)

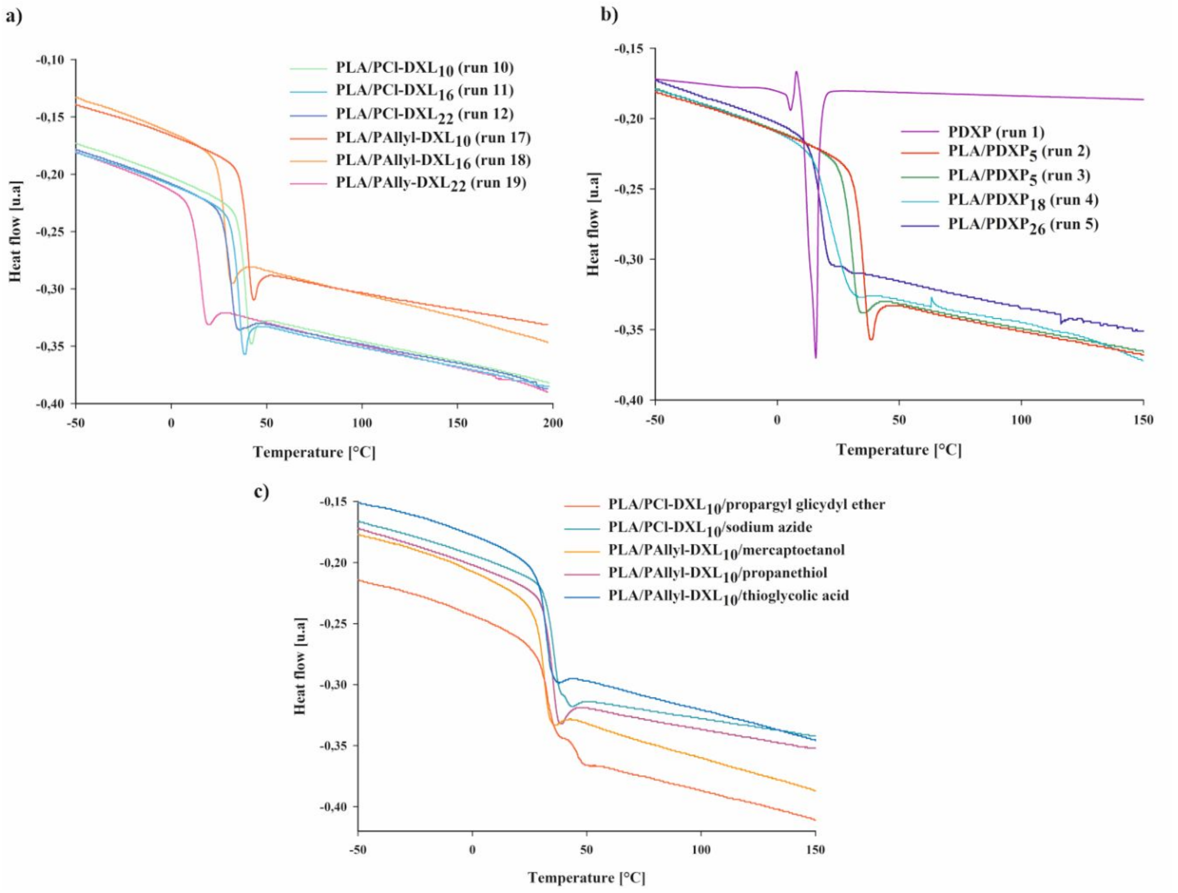

Figure S12. The DSC traces of a) PLA/PDXP, b) PLA/PCl-DXL or PLA/Pallyl-DXL copolymers, and c) DSC traces of copolymers after modification

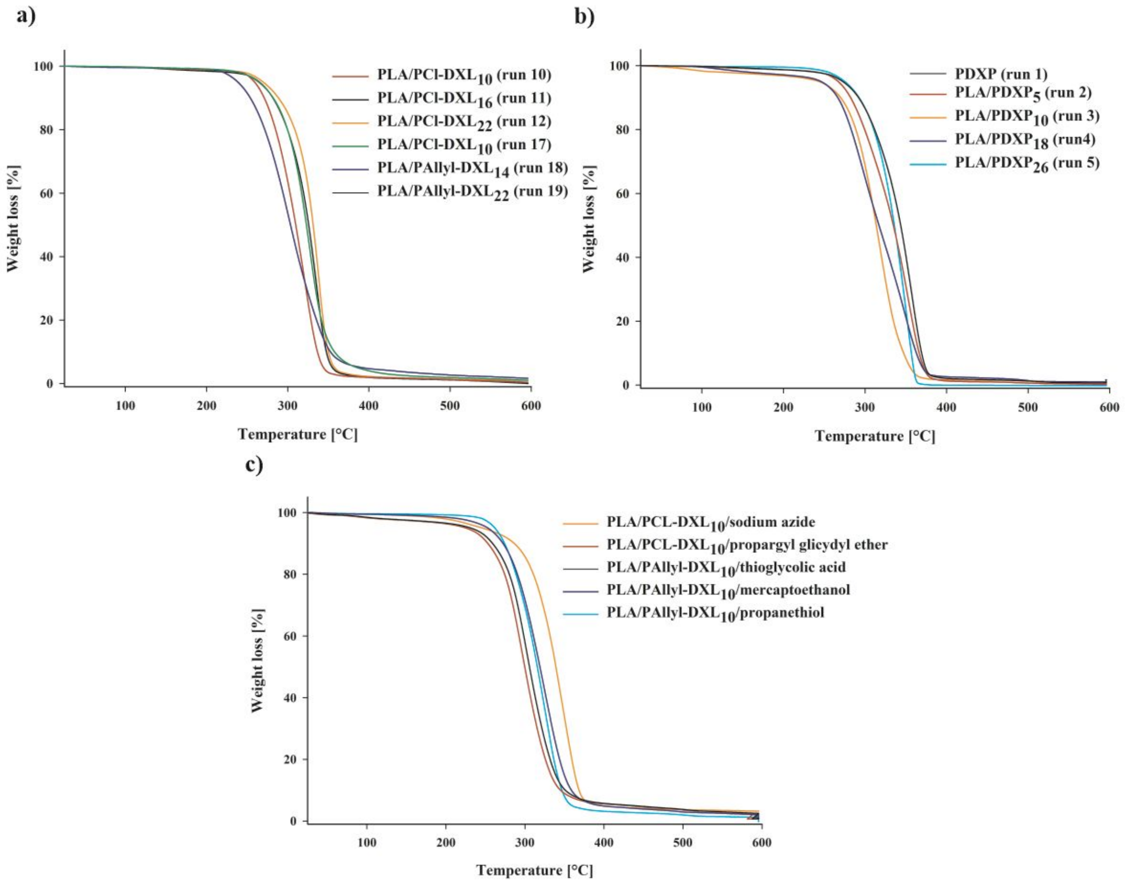

Figure S13. The TGA traces of a) PLA/PDXP, b) PLA/PCl-DXL or PLA/Pallyl-DXL copolymers, and c) DSC traces of copolymers after modification

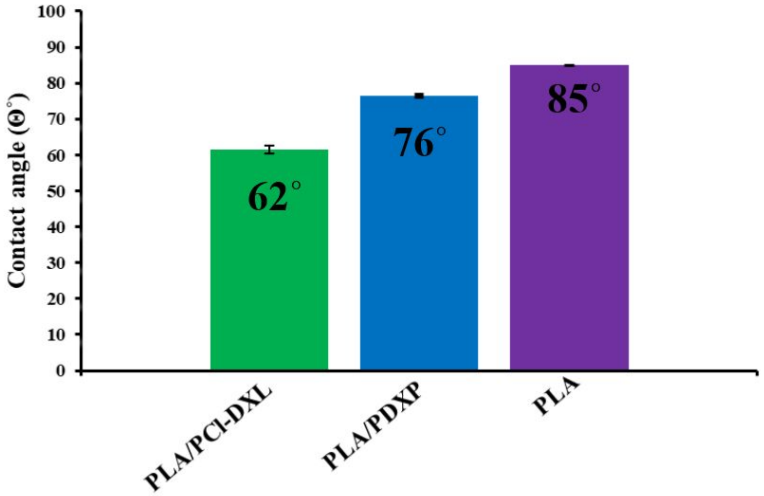

Figure S14. Contact angles measured for neat PLA and PLA/PDXL and PLA/PCl-DXL copolymers (films on a glass plate with water as a reference liquid)

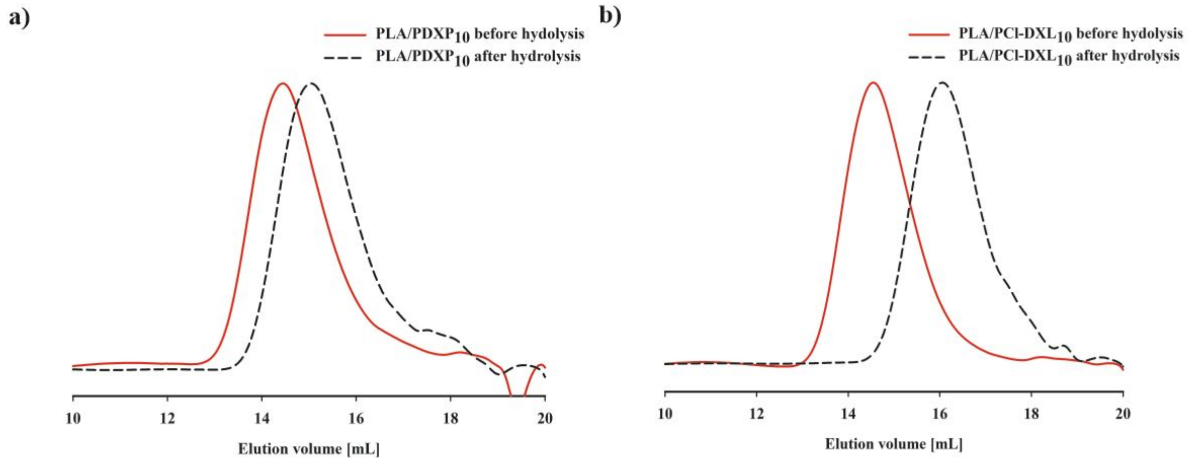

Figure S15. The GPC traces of a) PLA/PDXP and b) PLA/PCl-DXL copolymers before and after hydrolysis
